# Supplementary material for: Two-photon fluorescence imaging and specifically biosensing of norepinephrine on a 100-ms timescale
Source: Nat Commun. 2023 Mar 14;14:1419. doi: 10.1038/s41467-023-36869-3 (PMC10014876; doi:10.1038/s41467-023-36869-3)
Supplement: Supplementary file 1 — Supplementary Information [file 41467_2023_36869_MOESM1_ESM.pdf]

Supplementary Information

Two-photon fluorescence imaging and specifically biosensing of norepinephrine on a 100-ms timescale

Leiwen Mao, Yujie Han, Qi-Wei Zhang,* and Yang Tian*

Shanghai Key Laboratory of Green Chemistry and Chemical Processes, Department of Chemistry, School of Chemistry and Molecular Engineering, East China Normal University, Shanghai 200241, P.R. China.

***Corresponding Authors:**

qwzhang@chem.ecnu.edu.cn (Q.-W. Zhang); ytian@chem.ecnu.edu.cn (Y. Tian)

Contents

1. Syntheses.

2. Structural characterizations, optical measurements and cell experiments.

1. Syntheses.

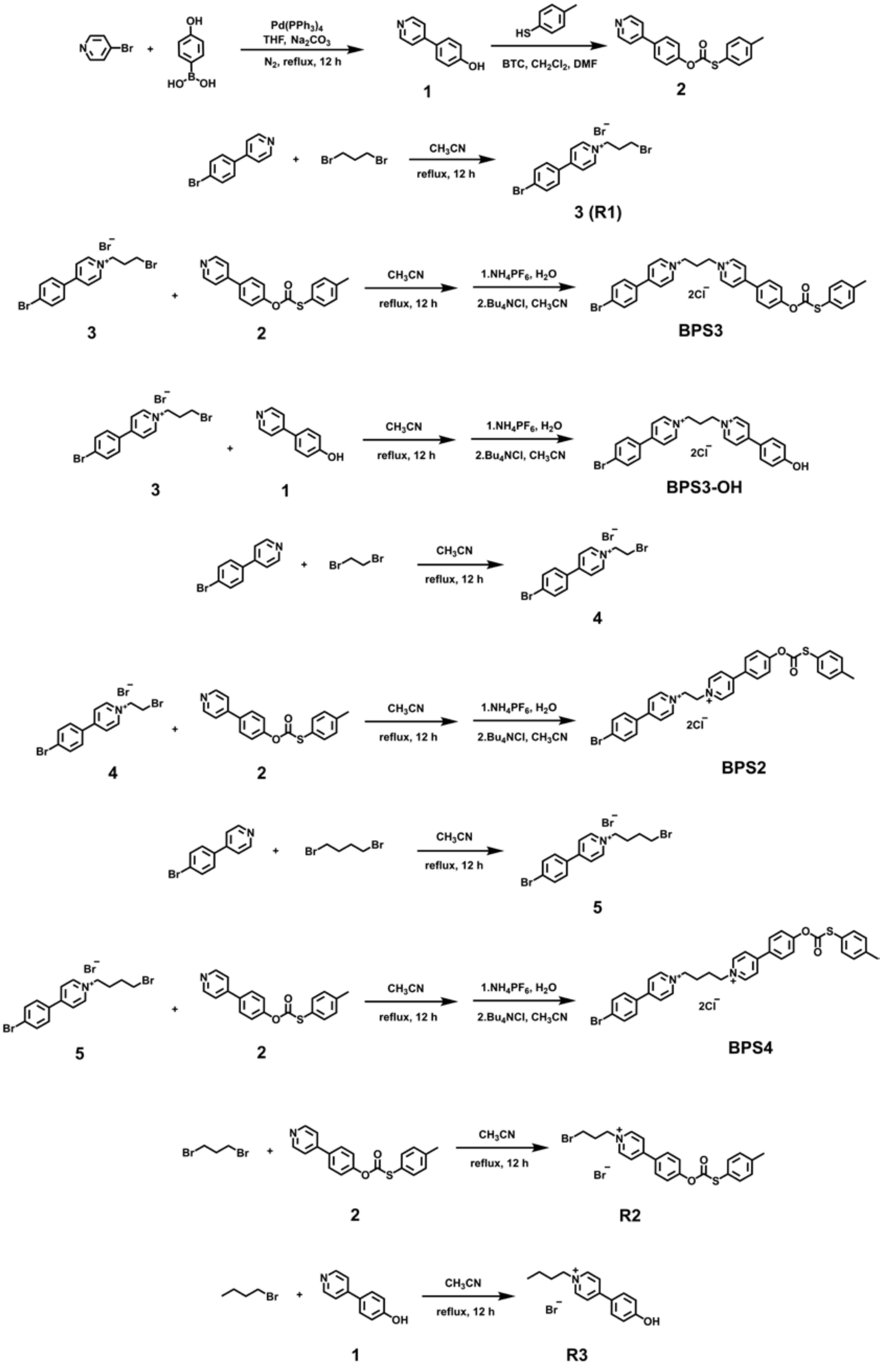

Fig. 1 Synthesis of probe BPS3 and reference compounds BPS2, BPS4, R2, and R3.

Synthesis of 4-(pyridin-4-yl)phenol (1).

$Pd(PPh_3)_4$ (80 mg, 0.07 mmol), 4-bromopyridine (0.56 g, 3.54 mmol) and (4-hydroxyphenyl) boronic acid (0.50 g, 3.62 mmol) in THF (30.0 mL) were mixed with a degassed aqueous solution of Na_2CO_3 (3.18 g, 30.0 mmol, in 15 mL H_2O). The resulting mixture was refluxed for 12 h under nitrogen. The product was extracted with ethyl acetate (50 mL × 3 times), and the organic layer was washed with saturated NaCl solution, which was dried with sodium sulfate. The solvent was removed in vacuum to get the crude product, which was further purified by column chromatography over silica gel (petroleum ether / ethyl acetate, 1/2), and compound 1 was obtained as light yellow solid (0.27 g, 45 %). ^{1}H NMR (500 MHz, Methanol-d_4) δ 8.49 (d, J = 6.4 Hz, 2H), 7.65 (d, J = 6.4 Hz, 2H), 7.62 (s, 2H), 6.92 (d, J = 8.7 Hz, 2H). ^{13}C NMR (126 MHz, Methanol-d_4) δ 158.99, 149.22, 148.88, 128.16, 127.94, 120.87, 115.67. HR-MS (ESI) m/z for $C_{11}H_9NO$ $[M+H]^+$ calcd. 172.0762, found: 172.0759.

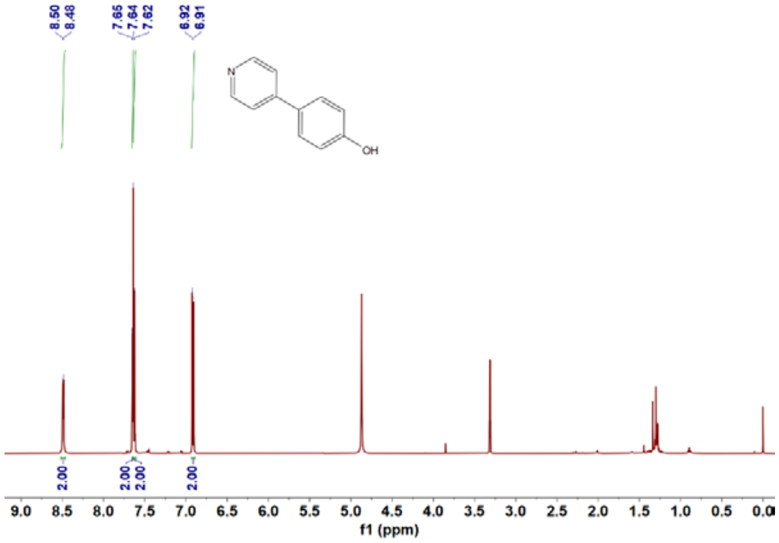

Fig. 2 ^{1}H NMR spectrum of compound 1 in methanol-d_4 (25°C).

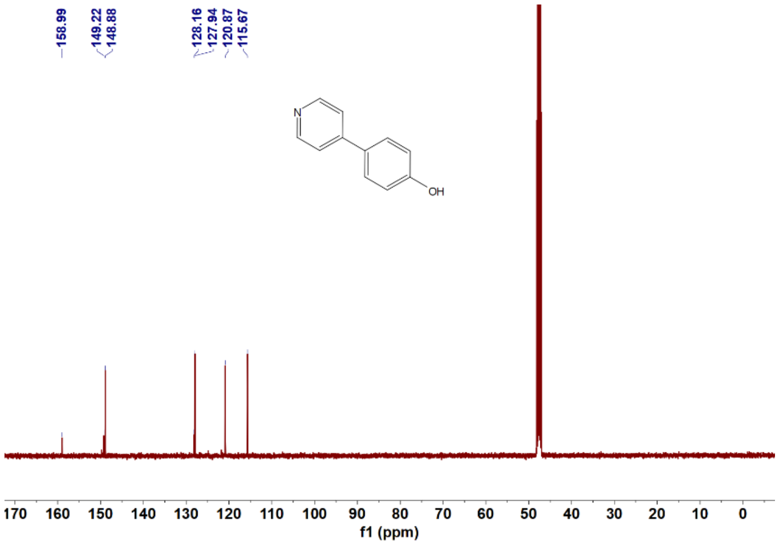

Fig. 3 ^{13}C NMR spectrum of compound 1 in methanol-d_4 (25°C).

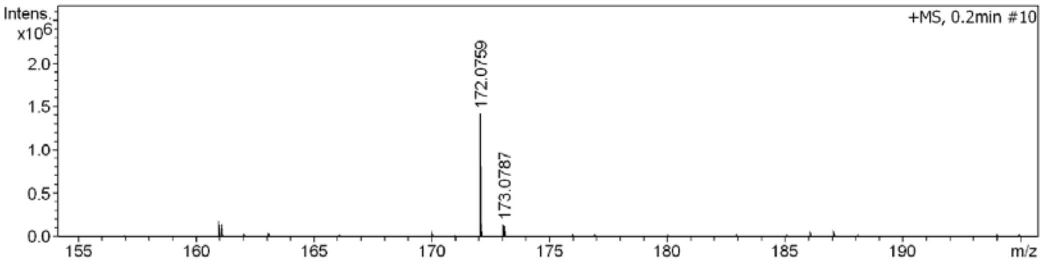

Fig. 4 ESI-HRMS spectrum of compound 1.

Synthesis of *O*-(4-(pyridin-4-yl)phenyl) *S*-(p-tolyl) carbonothioate (2).

Pyridine (10.00 mmol, in 5 mL CH_2Cl_2) was added dropwise to a solution of p-toluenethiol (1.24 g, 10.00 mmol) and triphosgene (BTC) (1.48 g, 5.00 mmol) in CH_2Cl_2 (20.0 mL) at 0°C. The mixture was stirred for 1 h at 0°C and then poured into 100.0 mL of ice water. The organic layer was separated, which was washed with H_2O, and dried with sodium sulfate. The crude product was obtained by removing the solvent under reduced pressure, which was used for the next step without purification. Compound 1 (0.17 g, 1.00 mmol) and triethylamine (0.20 g, 2.00 mmol) were dissolved in 10.0 mL DMF, and mixed with the previous crude product (400 mg in 5 mL CH_2Cl_2). The mixture was reacted for 10 h. After that, the solvent was removed under reduced pressure, and the residue was purified by column chromatography using petroleum ether / ethyl acetate (3/1) as eluent to give compound 2 as yellow solid (0.24 g, 74%). ^{1}H NMR (500 MHz, Chloroform-*d*) δ 8.67 (d, *J* = 5.3 Hz, 2H), 7.65 (d, *J* = 8.3 Hz, 2H), 7.54 (d, *J* = 5.3 Hz, 2H), 7.49 (d, *J* = 7.8 Hz, 2H), 7.33 (d, *J* = 8.3 Hz, 2H), 7.24 (s, 2H), 2.39 (s, 3H). ^{13}C NMR (126 MHz, Chloroform-*d*) δ 169.09, 152.10, 150.08, 147.52, 140.46, 135.98, 134.84, 130.20, 128.18, 123.36, 121.95, 121.63, 21.37. HR-MS (ESI) m/z for $C_{19}H_{15}NO_2S$ $[M+H]^+$ calcd. 322.0902, found: 322.0898.

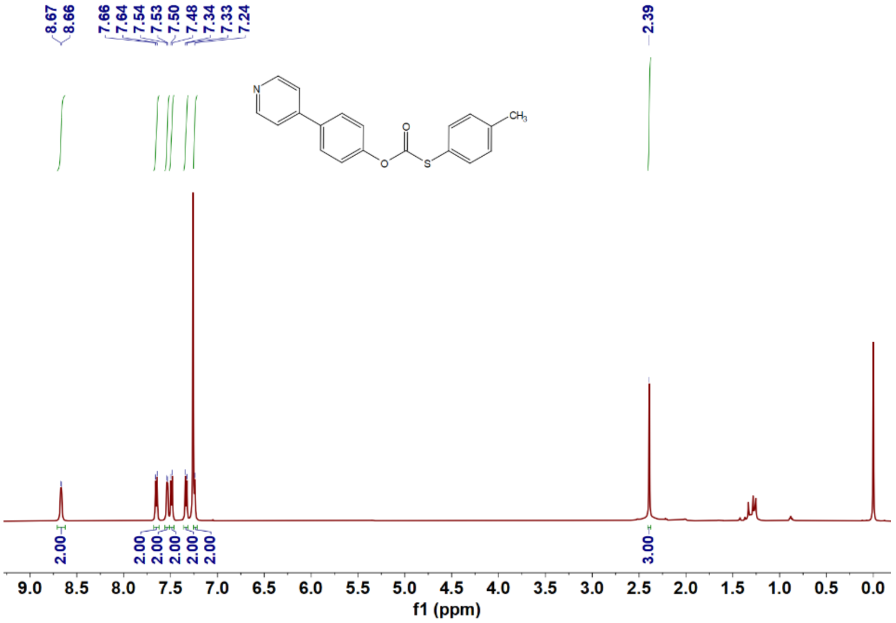

Fig. 5 ^{1}H NMR spectrum of compound 2 in chloroform-*d* (25°C).

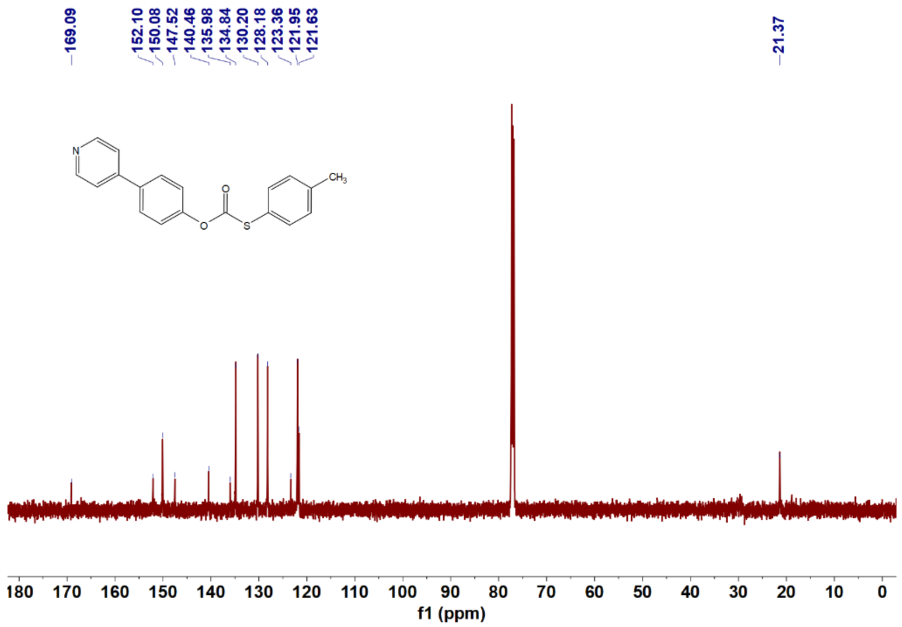

Fig. 6 ^{13}C NMR spectrum of compound 2 in chloroform-*d* (25°C).

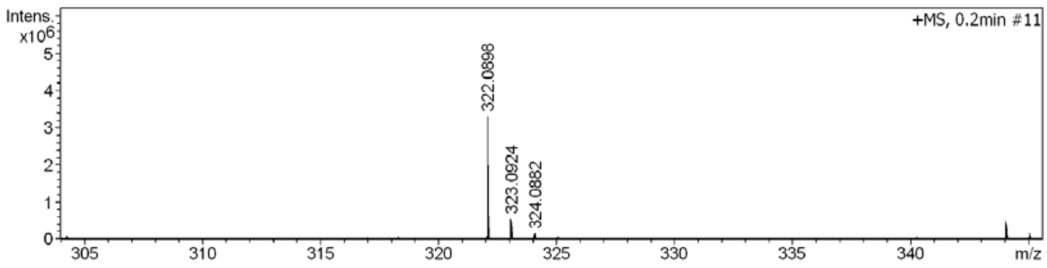

Fig. 7 ESI-HRMS spectrum of compound 2.

Synthesis of 4-(4-bromophenyl)-1-(3-bromopropyl)pyridin-1-ium (3) (also named as control compound R1).

4-(4-bromophenyl)pyridine (0.50 g, 2.14 mmol) and 1,3-dibromopropane (4.32 g, 21.40 mmol) were dissolved in 25 mL acetonitrile and reflexed for 12 h. After being cooled to room temperature, the reaction mixture was dispersed in 250 mL ethyl acetate. The mixture was filtered and the solid was washed with ethyl acetate to get compound 3 as white solid (0.79 g, 85%). ^{1}H NMR (500 MHz, DMSO-d_6) δ 9.16 (d, J = 7.0 Hz, 2H), 8.57 (d, J = 7.1 Hz, 2H), 8.05 (s, 2H), 7.88 (s, 2H), 4.73 (s, 2H), 3.61 (s, 2H), 2.55 (s, 2H). ^{13}C NMR (151 MHz, Methanol-d_4) δ 155.74, 144.84, 132.88, 132.80, 129.55, 126.97, 124.82, 59.20, 33.11, 27.81. HR-MS (ESI) m/z for $C_{14}H_{14}Br_3N$ [M-Br]$^+$, calcd. 355.9467, found: 355.9472.

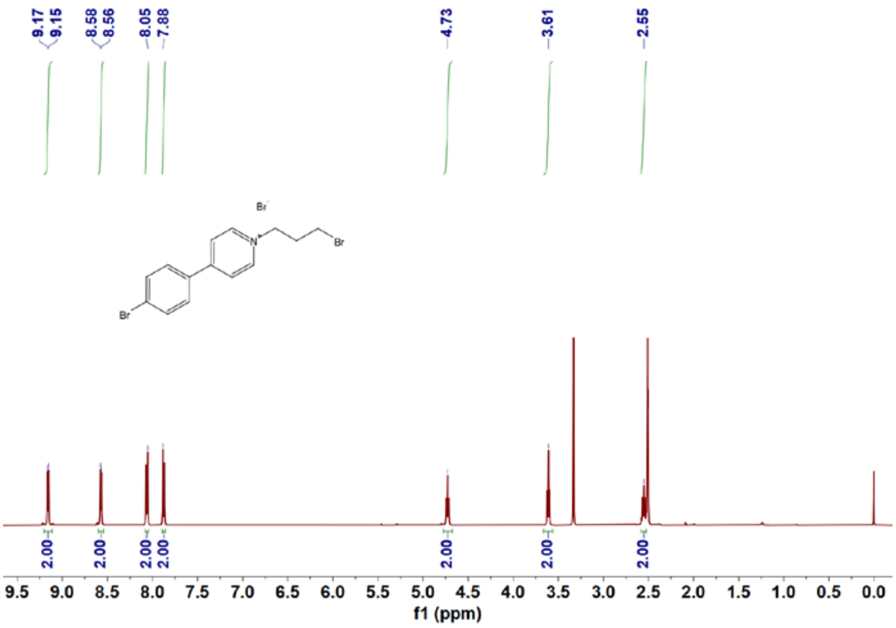

Fig. 8 ^{1}H NMR spectrum of compound 3 in DMSO-d_6 (25°C).

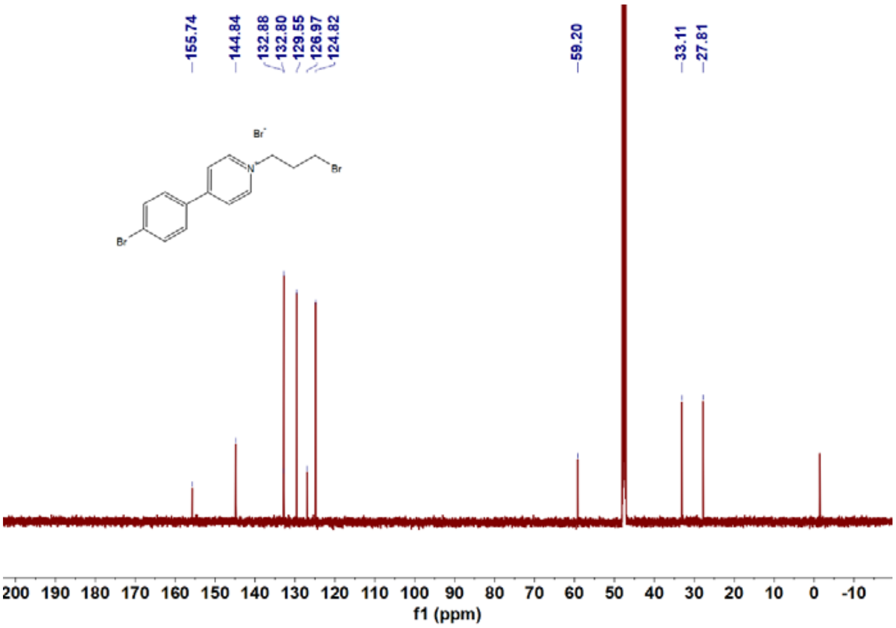

Fig. 9 ^{13}C NMR spectrum of compound 3 in methanol-d_4 (25°C).

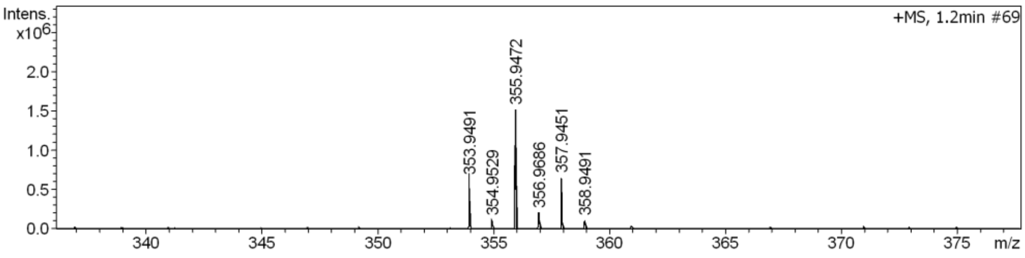

Fig. 10 ESI-HRMS spectrum of compound 3.

Synthesis of 1-(2-bromoethyl)-4-(4-bromophenyl)pyridin-1-ium (4).

4-(4-bromophenyl) pyridine (0.30 g, 1.28 mmol) and 1,2-dibromopropane (2.41 g, 12.80 mmol) were dissolved in 15 mL acetonitrile and reflexed for 12 h. After being cooled to room temperature, the reaction mixture was dispersed in 250 mL ethyl acetate. The mixture was filtered and the solid was washed with ethyl acetate to get compound 4 as white solid (0.46 g, 85%). ^{1}H NMR (500 MHz, DMSO-d_6) δ 9.17 (d, J = 7.1 Hz, 2H), 8.62 (d, J = 7.1 Hz, 2H), 8.07 (d, J = 8.7 Hz, 2H), 7.87 (d, J = 8.7 Hz, 2H), 5.05 (s, 2H), 4.14 (s, 2H). ^{13}C NMR (126 MHz, DMSO-d_6) δ 154.76, 145.74, 142.25, 133.17, 130.76, 126.99, 124.80, 60.68, 32.17. HR-MS (ESI) m/z for $C_{13}H_{12}Br_3N$ [M-Br]$^+$, calcd. 341.9311, found: 341.9303.

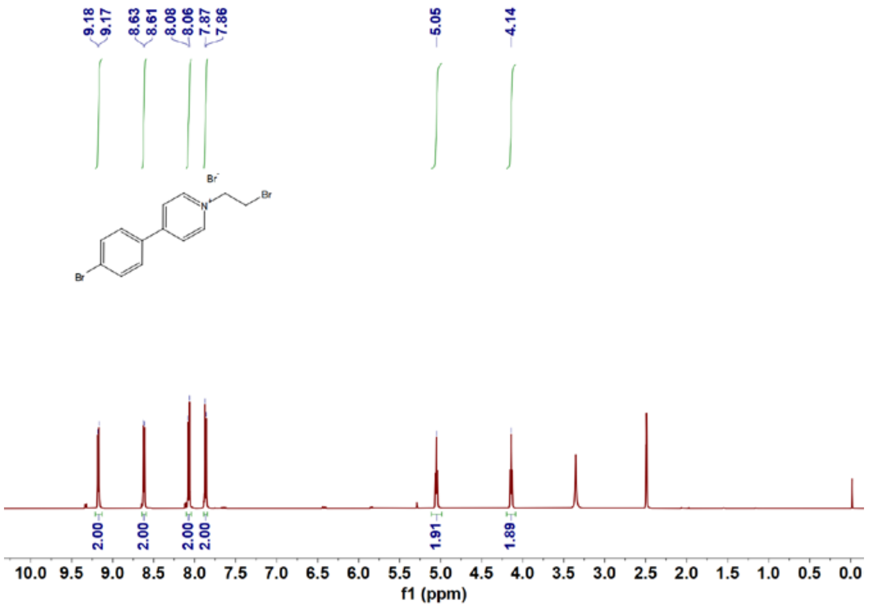

Fig. 11 ^{1}H NMR spectrum of compound 4 in DMSO-d_6 (25°C).

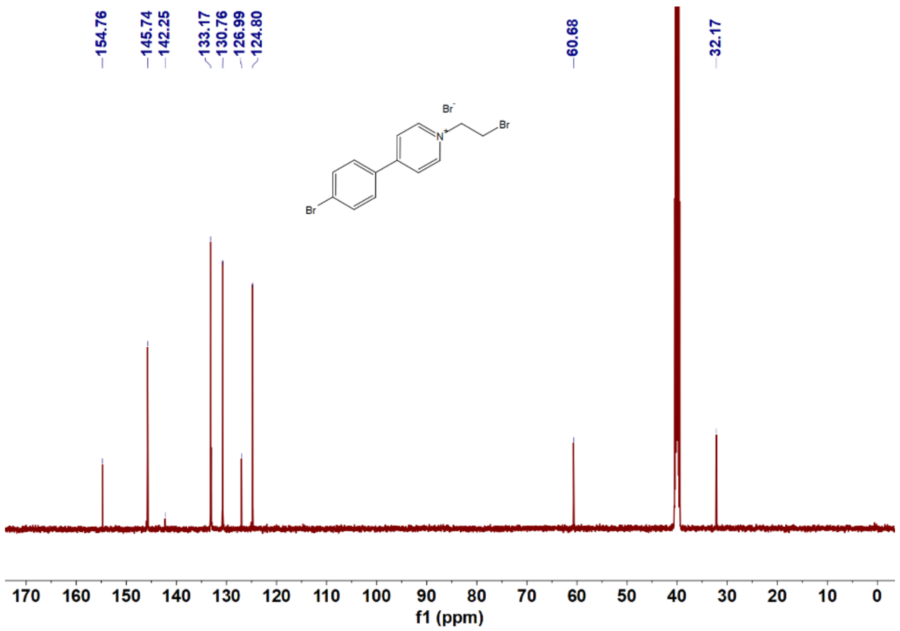

Fig. 12 ^{13}C NMR spectrum of compound 4 in DMSO-d_6 (25°C).

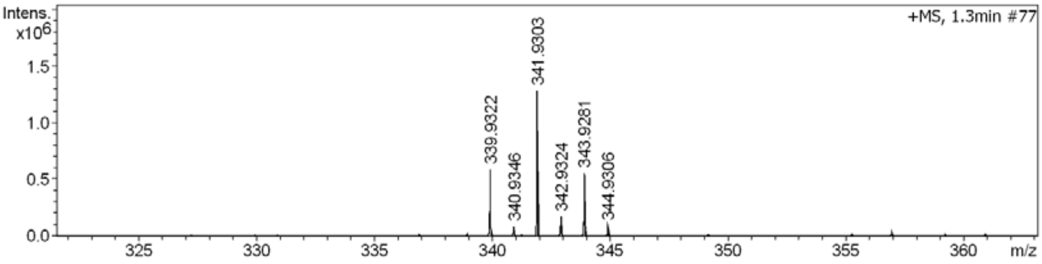

Fig. 13 ESI-HRMS spectrum of compound 4.

Synthesis of 1-(4-bromobutyl)-4-(4-bromophenyl)pyridin-1-ium (5).

4-(4-bromophenyl) pyridine (0.30 g, 1.28 mmol) and 1,4-dibromopropane (2.76 g, 12.80 mmol) were dissolved in 15.0 mL CH_3CN and reflexed for 12 h. After being cooled to room temperature, the reaction mixture was dispersed in 250 mL ethyl acetate. The mixture was filtered and the solid was washed with ethyl acetate to get compound 5 as white solid (0.49 g, 85%). ^{1}H NMR (500 MHz, DMSO-d_6) δ 9.14 (d, J = 7.1 Hz, 2H), 8.55 (d, J = 7.0 Hz, 2H), 8.04 (d, J = 8.7 Hz, 2H), 7.86 (d, J = 8.7 Hz, 2H), 4.63 (s, 2H), 3.58 (s, 2H), 2.05 (d, J = 7.7 Hz, 2H), 1.92 – 1.76 (m, 2H). ^{13}C NMR (126 MHz, DMSO-d_6) δ 154.07, 145.37, 133.25, 133.13, 130.64, 126.72, 125.09, 59.55, 34.49, 29.90, 29.17. HR-MS (ESI) m/z for $C_{15}H_{16}Br_3N$ [M-Br]$^+$ calcd. 369.9624, found: 369.9621.

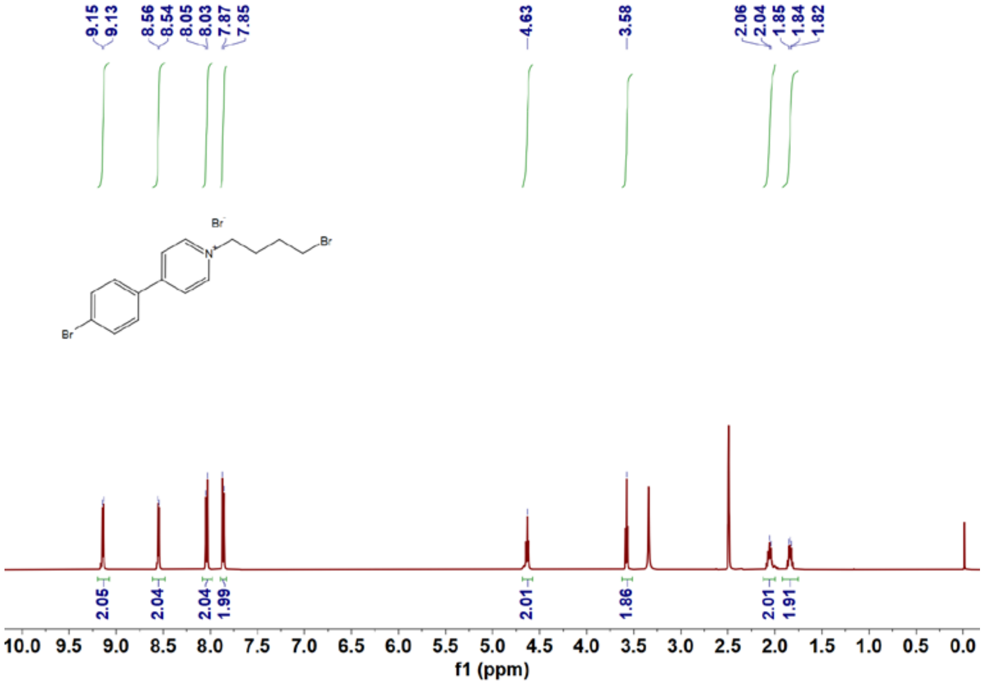

Fig. 14 ^{1}H NMR spectrum of compound 5 in DMSO-d_6 (25°C).

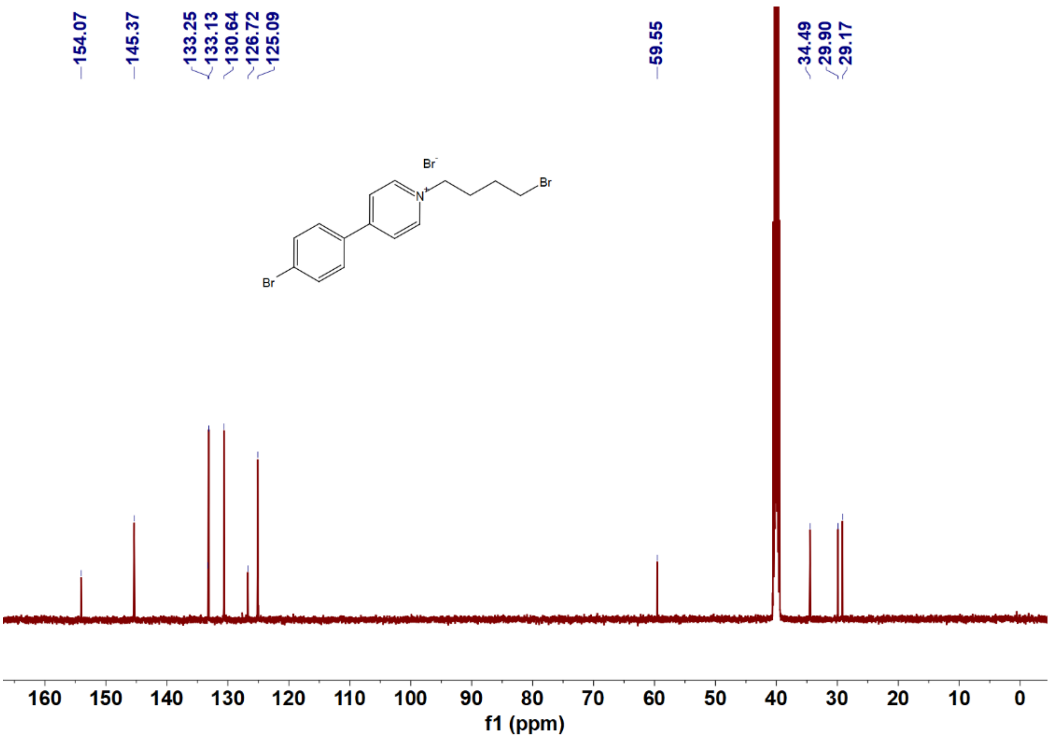

Fig. 15 ^{13}C NMR spectrum of compound 5 in DMSO-d_6 (25°C).

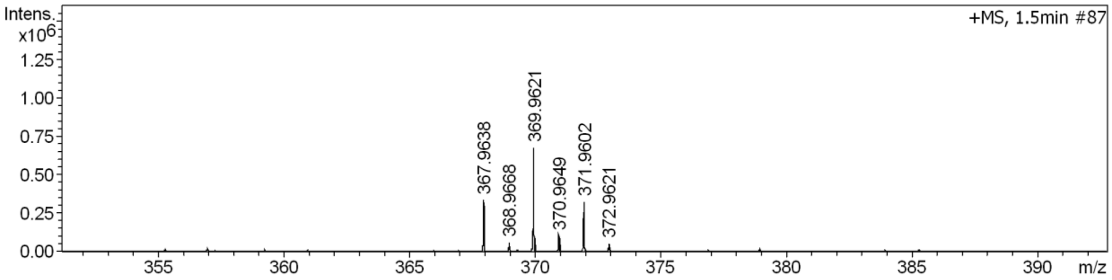

Fig. 16 ESI-HRMS spectrum of compound 5.

Synthesis of 4-(4-bromophenyl)-1-(3-(4-(4-(((p-tolylthio)carbonyl)oxy)phenyl)pyridin-1-ium-1-yl)propyl)pyridin-1-ium (BPS3).

Compound 3 (0.20 g, 0.46 mmol) and compound 2 (0.16 g, 0.51 mmol) were dissolved in 10 mL acetonitrile and refluxed for 12 h. After that, the mixture was cooled to room temperature and the solvent was removed by evaporation. The resulting residue was washed with ethyl acetate and dried. BPS3 was obtained as yellow solid after ion exchange (0.13 g, 42 %). ^{1}H NMR (500 MHz, Methanol-d_4) δ 9.12 – 9.08 (m, 4H), 8.50 – 8.46 (m, 4H), 8.11 (d, J = 8.9 Hz, 2H), 7.96 (d, J = 8.7 Hz, 2H), 7.83 (d, J = 8.7 Hz, 2H), 7.51 (dd, J = 11.6, 8.5 Hz, 4H), 7.32 (d, J = 7.9 Hz, 2H), 4.89 (s, 4H), 2.86 (s, 2H), 2.41 (s, 3H). ^{13}C NMR (126 MHz, Methanol-d_4) δ 168.67, 155.86, 154.52, 144.82, 144.73, 140.62, 134.66, 132.83, 131.64, 129.85, 129.57, 129.54, 127.07, 124.90, 123.07, 122.50, 57.26, 57.19, 31.96, 19.90. HR-MS (ESI) m/z for $C_{33}H_{29}BrCl_2N_2O_2S$ [M-2Cl]$^{2+}$ calcd. 298.0561, found: 298.0558.

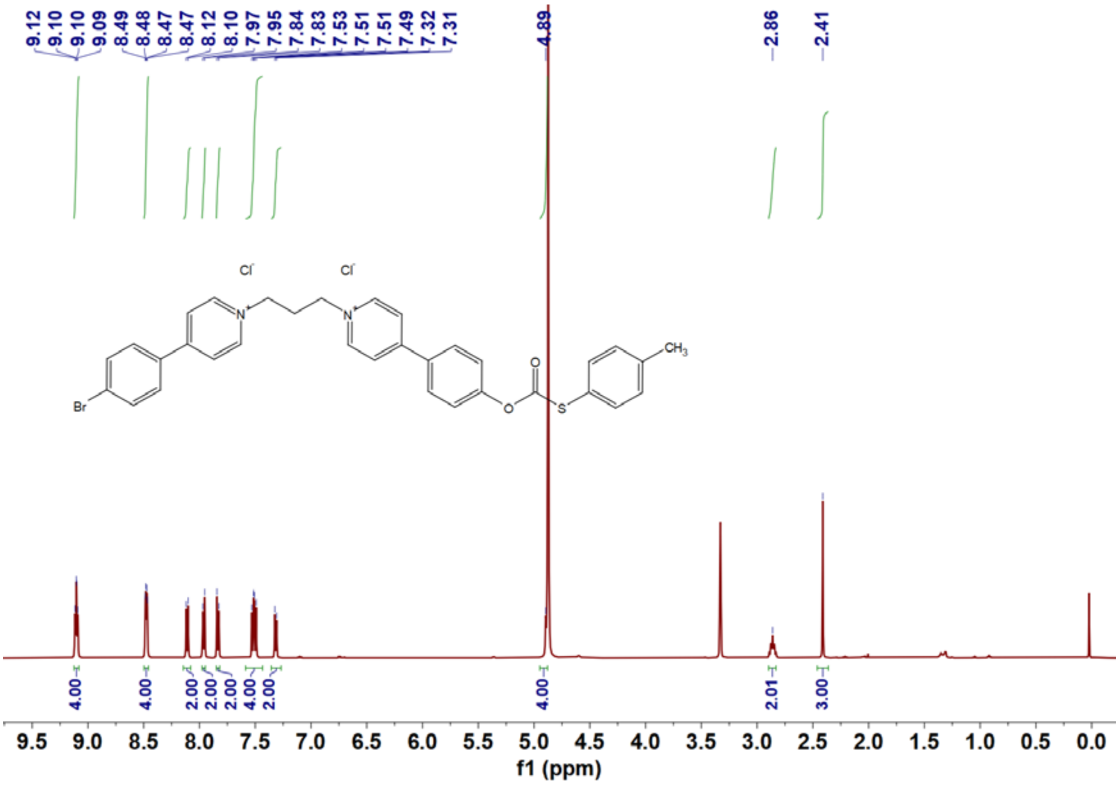

Fig. 17 ^{1}H NMR spectrum of BPS3 in methanol-d_4 (25°C).

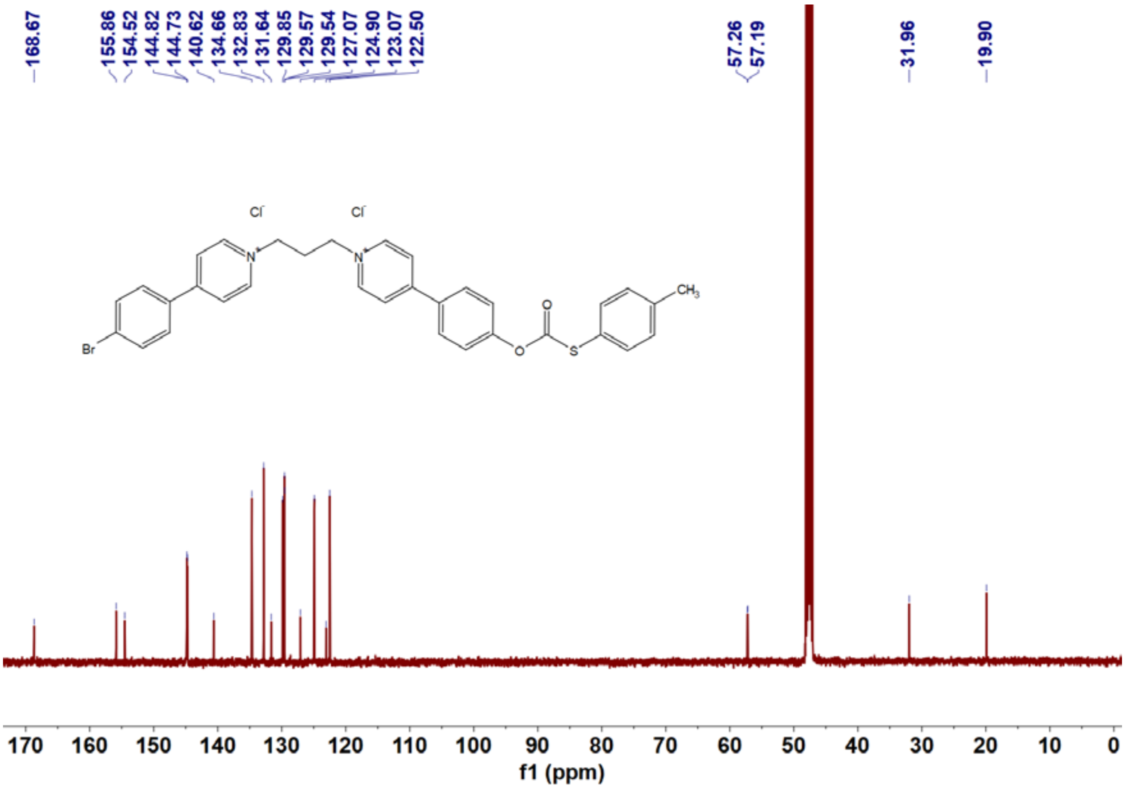

Fig. 18 ^{13}C NMR spectrum of BPS3 in methanol-d_4 (25°C).

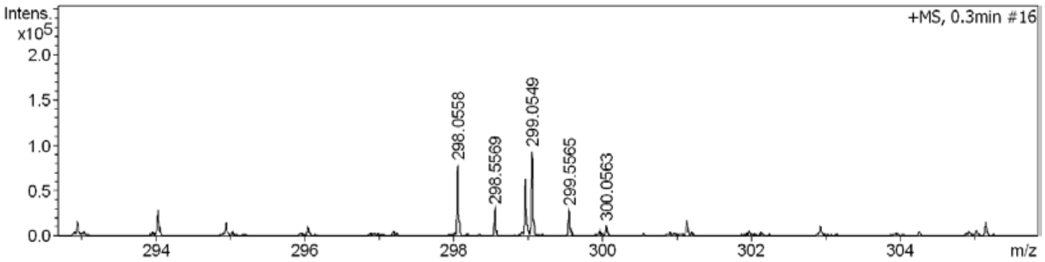

Fig. 19 ESI-HRMS spectrum of BPS3.

Synthesis of 4-(4-bromophenyl)-1-(3-(4-(4-hydroxyphenyl)pyridin-1-ium-1-yl)propyl)pyridin-1-ium (BPS3-OH).

Compound 3 (0.20 g, 0.46 mmol) and compound 1 (0.09 g, 0.51 mmol) were dissolved in 10 mL acetonitrile and refluxed for 12h. The mixture was cooled to room temperature and the solvent was removed by evaporation. The resulting residue was washed with ethyl acetate and dried. Product BPS3-OH was obtained as yellow solid after ion exchange (0.11 g, 45 %). ^{1}H NMR (500 MHz, Methanol-d_4) δ 9.08 (d, J = 7.0 Hz, 2H), 8.89 (d, J = 7.1 Hz, 2H), 8.48 (d, J = 7.0 Hz, 2H), 8.36 (d, J = 7.1 Hz, 2H), 7.97 (t, J = 8.7 Hz, 4H), 7.84 (d, J = 8.7 Hz, 2H), 7.03 (d, J = 8.9 Hz, 2H), 4.84 (d, J = 8.1 Hz, 2H), 4.80 – 4.75 (m, 2H), 2.82 (s, 2H). ^{13}C NMR (126 MHz, Methanol-d_4) δ 162.52, 144.78, 143.97, 132.84, 132.76, 129.94, 129.55, 127.11, 124.90, 123.96, 123.03, 116.52, 57.32, 56.63, 31.86. HR-MS (ESI) m/z for $C_{25}H_{23}BrCl_2N_2O$ [M-2Cl]$^{2+}$ calcd. 223.0491, found: 223.0489.

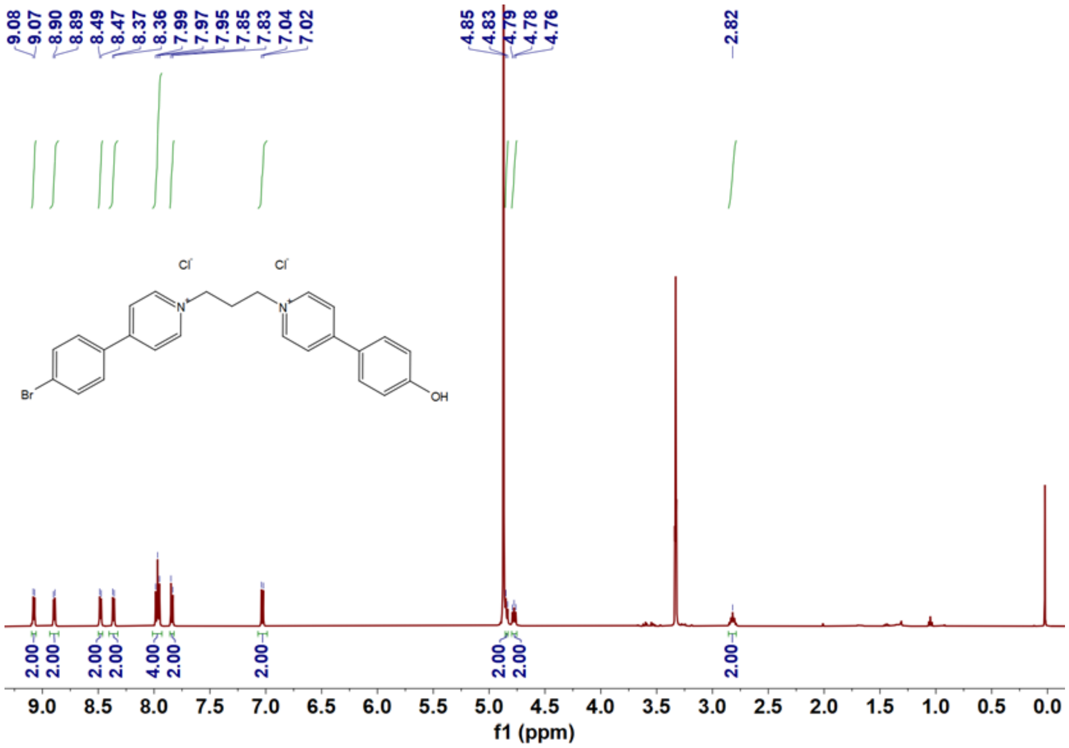

Fig. 20 ^{1}H NMR spectrum of BPS3-OH in methanol-d_4 (25°C).

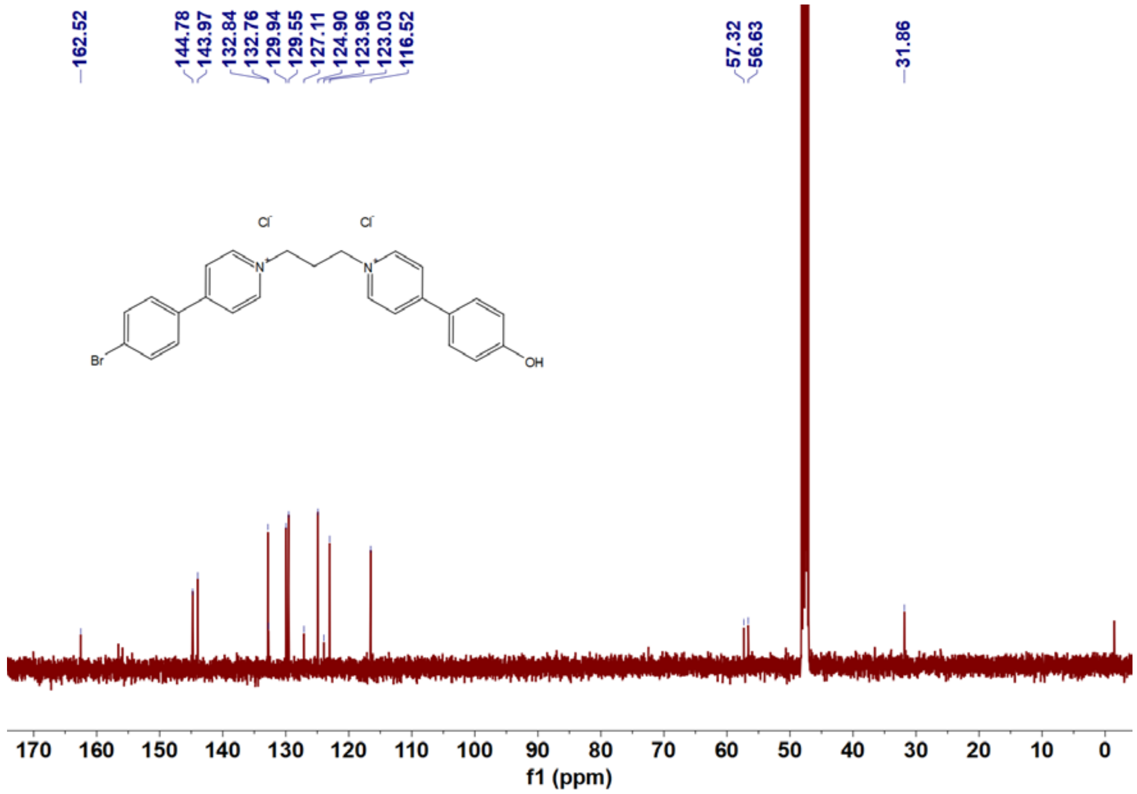

Fig. 21 ^{13}C NMR spectrum of BPS3-OH in methanol-d_4 (25°C).

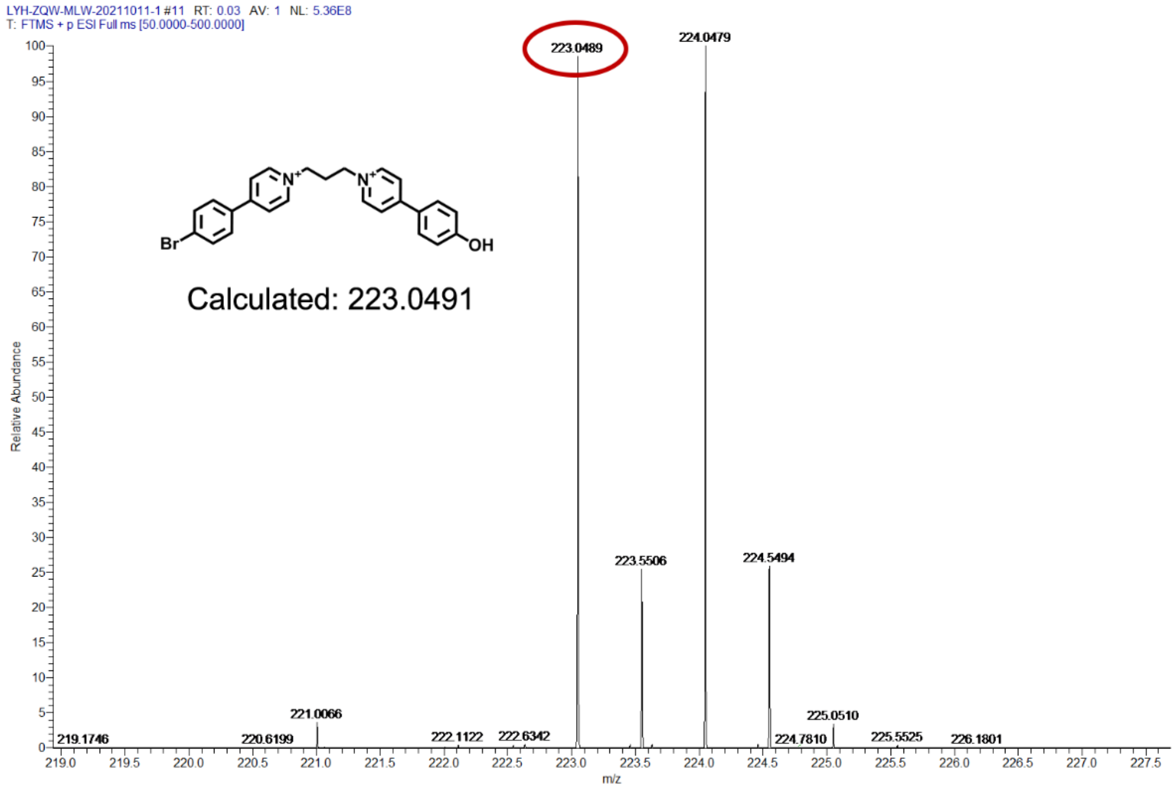

Fig. 22 ESI-HRMS spectrum of BPS3-OH.

Synthesis of 4-(4-bromophenyl)-1-(2-(4-(4-(((p-tolylthio)carbonyl)oxy)phenyl)pyridin-1-ium-1-yl)ethyl)pyridin-1-ium (BPS2).

Compound 4 (0.18 g, 0.42 mmol) and compound 2 (0.15 g, 0.47 mmol) were dissolved in 25.0 mL acetonitrile and refluxed for 12 h. The mixture was cooled to room temperature and the solvent was removed by evaporation. The resulting residue was washed with ethyl acetate and dried. Product BPS2 was obtained as light yellow solid after ion exchange (0.12 g, 42 %). ^{1}H NMR (500 MHz, Methanol-d_4) δ 9.07 – 9.03 (m, 4H), 8.50 (dd, J = 6.7, 2.7 Hz, 4H), 8.13 (d, J = 8.8 Hz, 2H), 7.99 – 7.97 (m, 2H), 7.85 – 7.83 (m, 2H), 7.59 – 7.42 (m, 4H), 7.30 (d, J = 7.8 Hz, 2H), 5.34 (s, 4H), 2.40 (s, 3H). ^{13}C NMR (126 MHz, Methanol-d_4) δ 168.66, 156.68, 154.75, 145.16, 140.64, 134.66, 132.88, 132.57, 131.42, 129.85, 129.74, 129.72, 127.44, 125.18, 125.15, 123.04, 122.55, 58.96, 58.92, 58.87, 19.90. HR-MS (ESI) m/z for $C_{32}H_{27}BrCl_2N_2O_2S$ [M-2Cl]$^{2+}$ calcd. 292.0473, found: 292.0469.

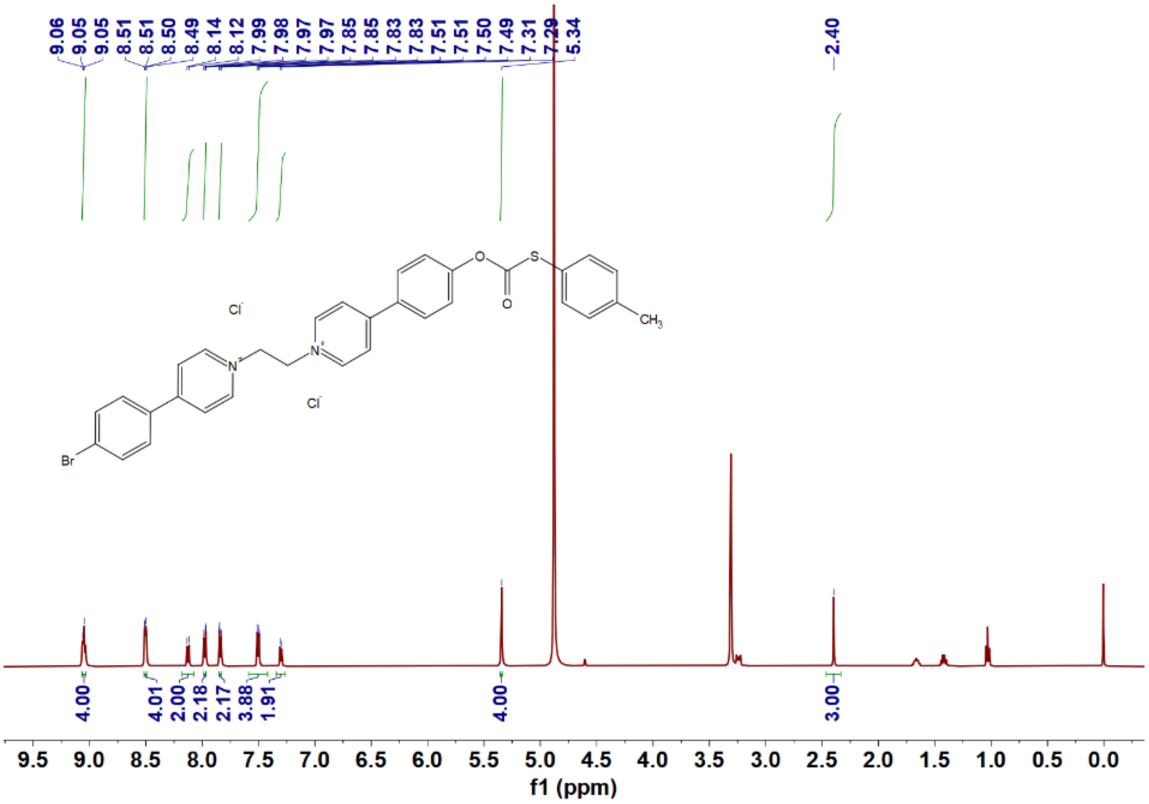

Fig. 23 ^{1}H NMR spectrum of BPS2 in methanol-d_4 (25°C).

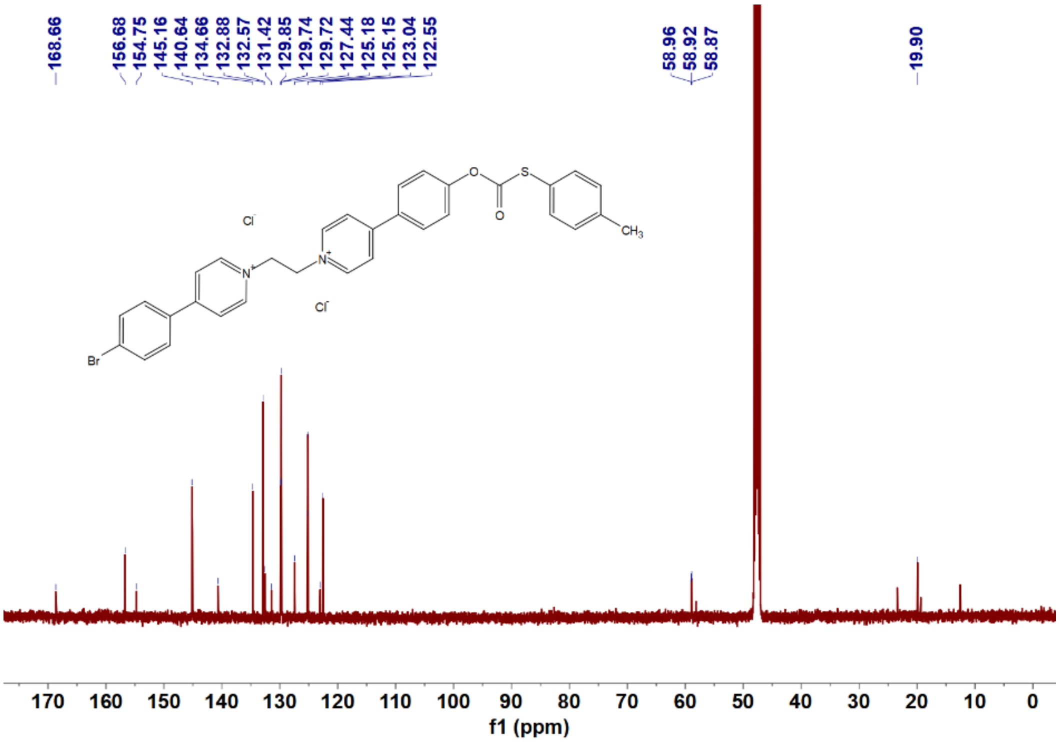

Fig. 24 ^{13}C NMR spectrum of BPS2 in methanol-d_4 (25°C).

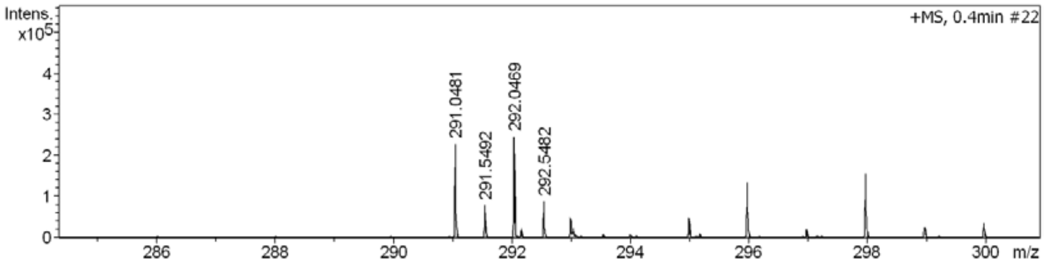

Fig. 25 ESI-HRMS spectrum of BPS2.

Synthesis of 4-(4-bromophenyl)-1-(4-(4-(4-(((p-tolylthio)carbonyl)oxy)phenyl)pyridin-1-ium-1-yl)butyl)pyridin-1-ium (BPS4).

Compound 5 (0.19 g, 0.42 mmol) and compound 2 (0.15 g, 0.47 mmol) were dissolved in 25 mL CH_3CN and refluxed for 12 h. The residue was cooled to room temperature and the solvent was removed by evaporation. The resulting mixture was washed with ethyl acetate and dried. Product was obtained as light yellow solid after ion exchange (0.12 g, 42 %). ^1H NMR (500 MHz, Methanol-d_4) δ 9.05 – 9.01 (m, 4H), 8.44 – 8.41 (m, 4H), 8.09 (d, J = 8.8 Hz, 2H), 7.95 – 7.93 (m, 2H), 7.82 (d, J = 8.7 Hz, 2H), 7.49 (dd, J = 12.0, 8.5 Hz, 4H), 7.30 (d, J = 7.8 Hz, 2H), 4.74 (s, 4H), 2.40 (s, 3H), 2.19 (s, 4H). ^{13}C NMR (126 MHz, Methanol-d_4) δ 168.67, 155.57, 154.44, 144.68, 144.59, 140.61, 134.66, 132.87, 132.79, 131.74, 129.84, 129.53, 129.49, 126.93, 124.82, 124.78, 123.08, 122.48, 59.82, 59.75, 27.49, 19.90. HR-MS (ESI) m/z for $C_{34}H_{31}BrCl_2N_2O_2S$ [M-2Cl]$^{2+}$ calcd. 306.0629, found: 306.0629.

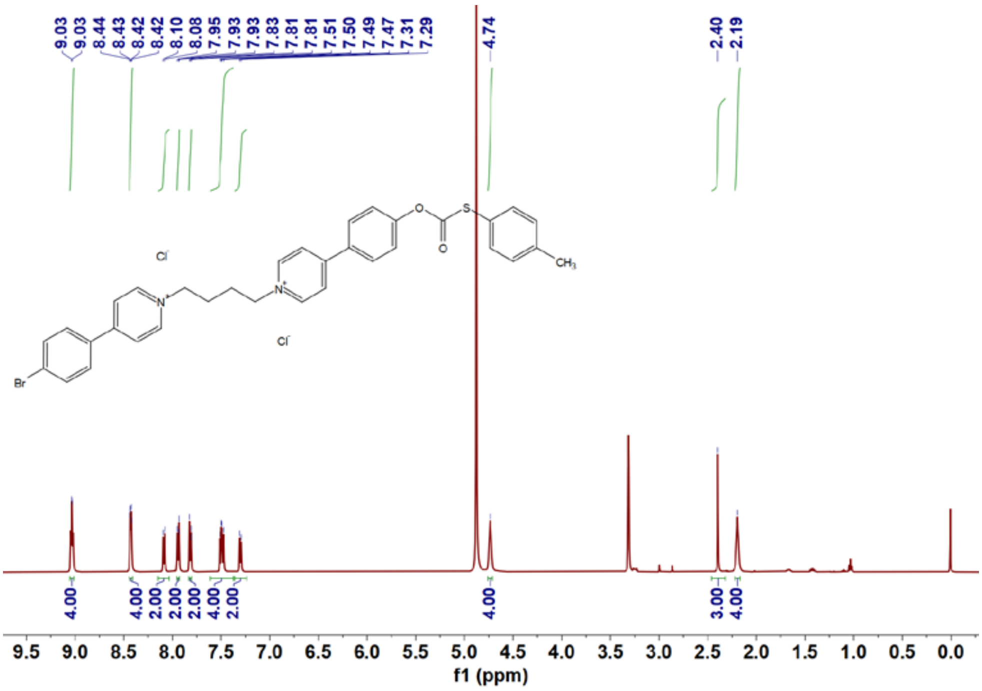

Fig. 26 ^{1}H NMR spectrum of BPS4 in methanol-d_4 (25°C).

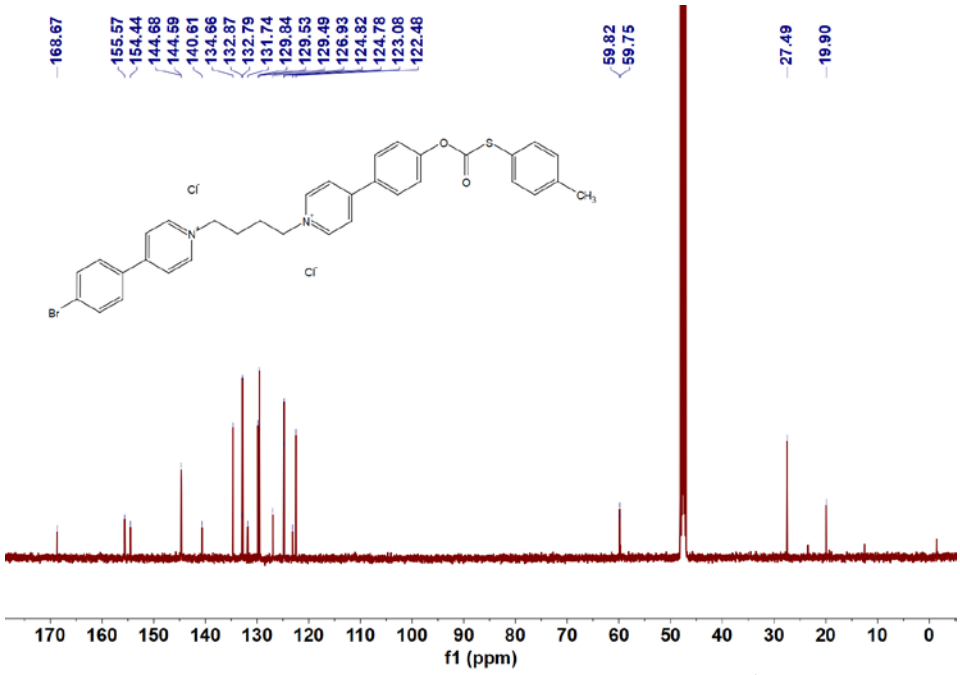

Fig. 27 ^{13}C NMR spectrum of BPS4 in methanol-d_4 (25°C).

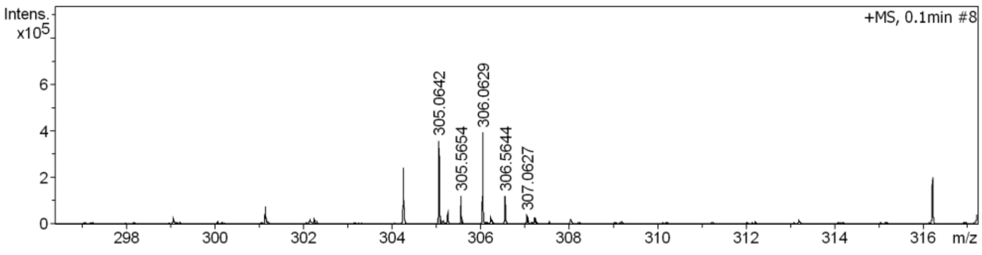

Fig. 28 ESI-HRMS spectrum of BPS4.

Synthesis of 1-(3-bromopropyl)-4-(4-(((p-tolylthio)carbonyl)oxy)phenyl)pyridin-1-ium (R2). Compound **2** (0.50 g, 1.56 mmol) and 1,3-dibromopropane (3.15 g, 15.6 mmol) were dissolved in 30 mL acetonitrile and refluxed for 12 h. The solvent and excess 1,3-dibromopropane were removed by evaporation. Product was obtained as yellow solid (0.37 g, 45%). ^{1}H NMR (500 MHz, Methanol-d_4) δ 8.99 (d, J = 7.1 Hz, 2H), 8.33 (d, J = 7.1 Hz, 2H), 8.03 (d, J = 8.9 Hz, 2H), 7.50 (d, J = 8.2 Hz, 2H), 7.42 (d, J = 8.8 Hz, 2H), 7.27 (d, J = 8.1 Hz, 2H), 4.61 (t, J = 7.6 Hz, 2H), 2.35 (s, 3H), 1.99 (t, J = 7.6 Hz, 2H), 1.47 – 1.39 (m, 2H). ^{13}C NMR (126 MHz, Methanol-d_4) δ 168.78, 154.73, 154.20, 144.52, 140.61, 134.75, 131.61, 130.01, 129.61, 124.69, 123.08, 122.49, 60.60, 32.98, 20.14, 19.12. HR-MS (ESI) m/z for $C_{22}H_{21}Br_2NO_2S$ [M-Br]$^+$, calcd. 442.0471, found: 442.0479.

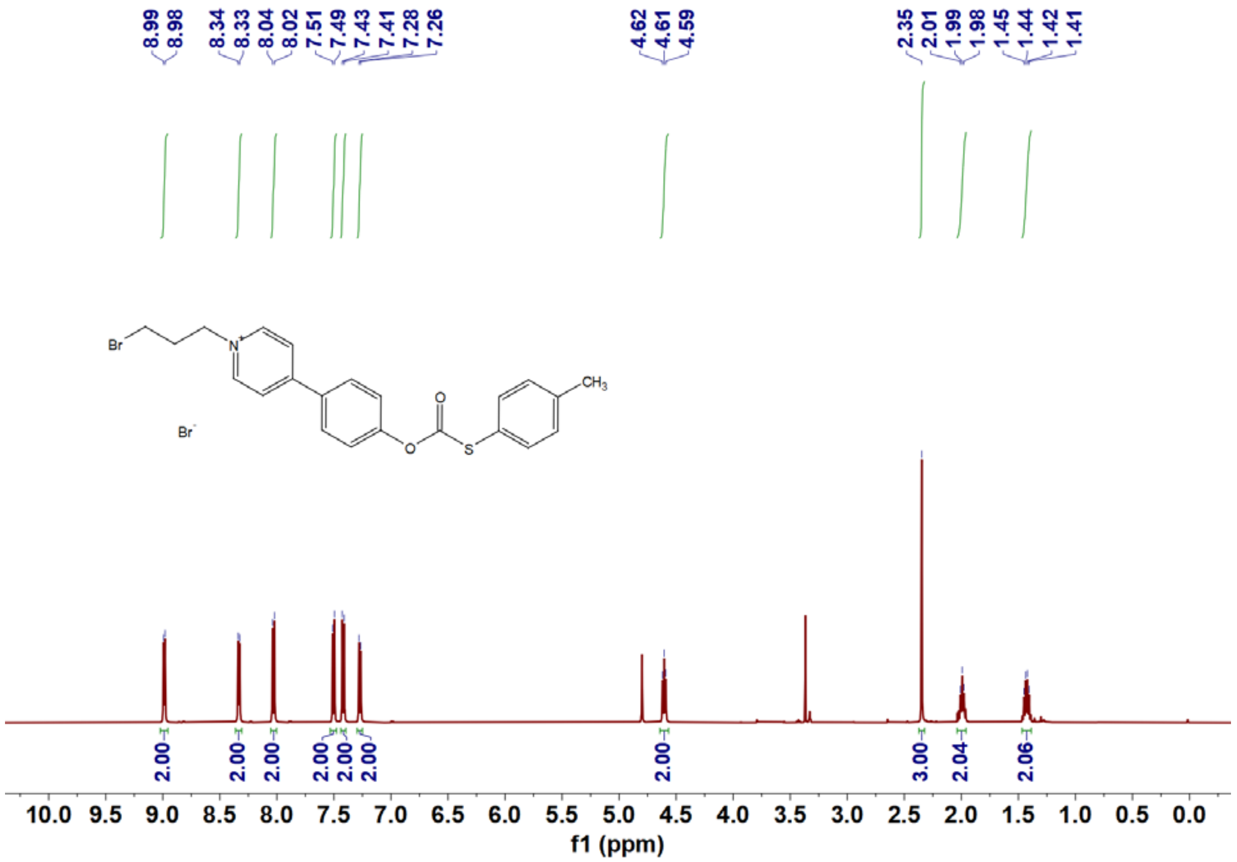

Fig. 29 ^{1}H NMR spectrum of compound R2 in methanol-d_4 (25°C).

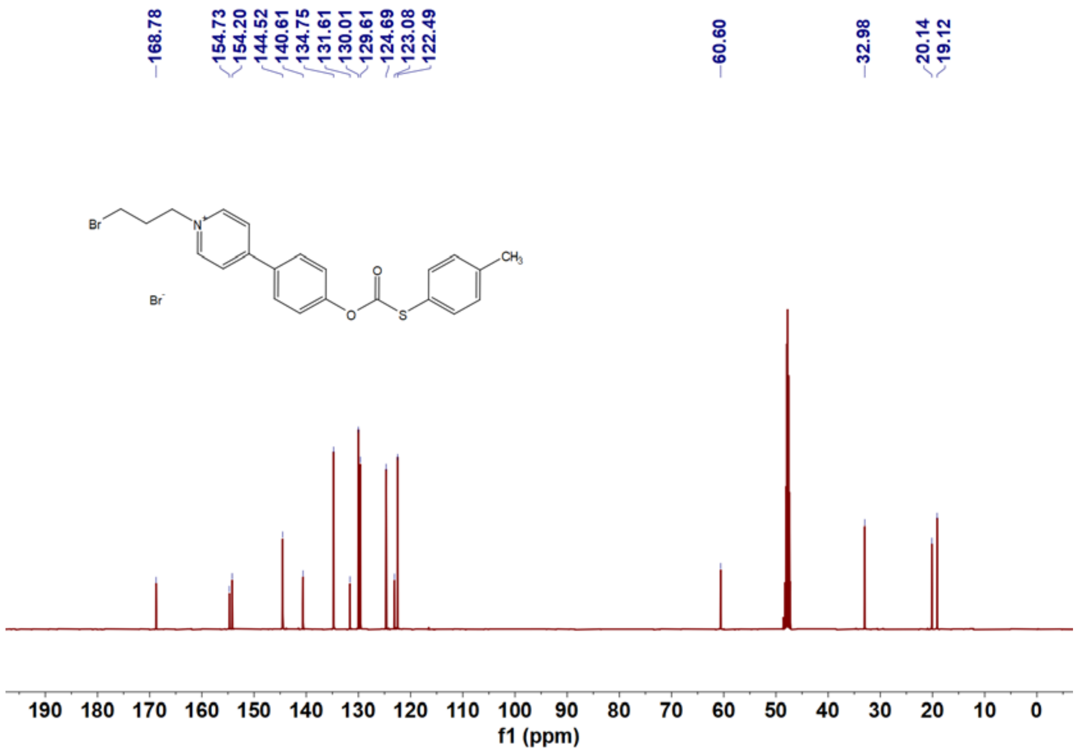

Fig. 30 ^{13}C NMR spectrum of compound R2 in methanol-d_4 (25°C).

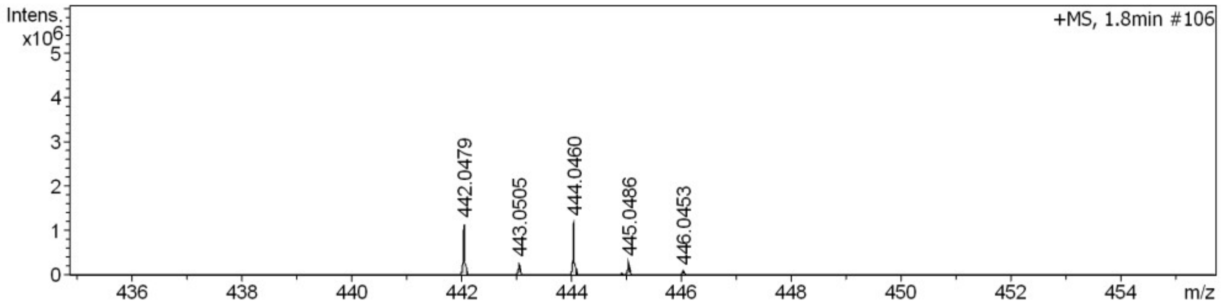

Fig. 31 ESI-HRMS spectrum of compound **R2**.

Synthesis of 1-butyl-4-(4-hydroxyphenyl)pyridin-1-ium (R3).

Compound **1** (0.50 g, 2.92 mmol) and 1-bromobutane (4.0 g, 29.2 mmol) were dissolved in 30 mL acetonitrile and refluxed for 12 h. After being cooled to room temperature, the reaction mixture was dispersed in 250 mL ethyl acetate. The mixture was filtered and the solid was washed with ethyl acetate to get compound R3 as white solid (0.38 g, 42%). ^{1}H NMR (500 MHz, Methanol-d_4) δ 8.83 (d, J = 7.1 Hz, 2H), 8.31 (d, J = 7.1 Hz, 2H), 7.96 (d, J = 8.9 Hz, 2H), 7.03 (d, J = 8.8 Hz, 2H), 4.57 (t, J = 7.5 Hz, 2H), 2.02 (s, 2H), 1.46 (d, J = 7.7 Hz, 2H), 1.04 (s, 3H). ^{13}C NMR (126 MHz, Methanol-d_4) δ 162.21, 156.04, 143.80, 129.80, 124.18, 122.89, 116.45, 60.04, 32.84, 19.04, 12.36. HR-MS (ESI) m/z for $C_{15}H_{18}BrNO$ [M-Br]$^+$, calcd. 228.1383, found: 228.1389.

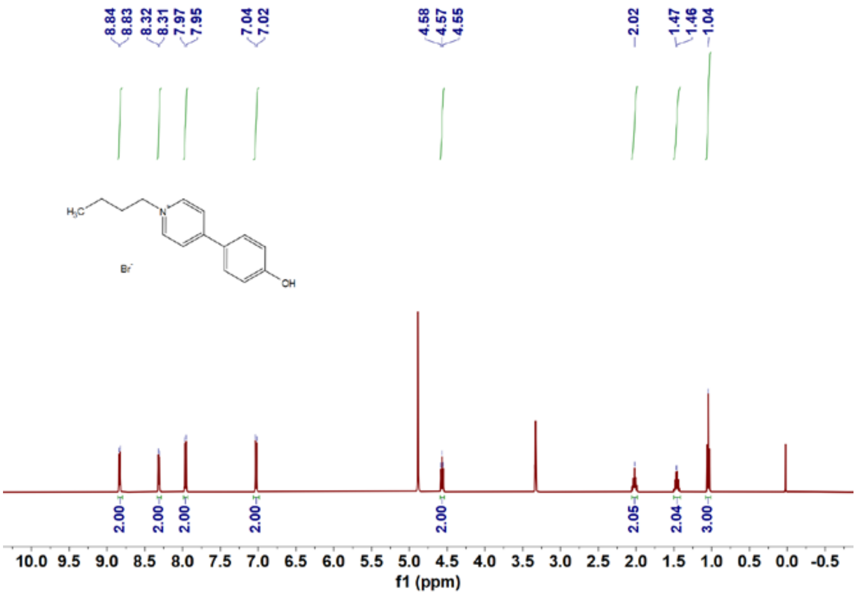

Fig. 32 ^{1}H NMR spectrum of compound R3 in methanol-d_4 (25°C).

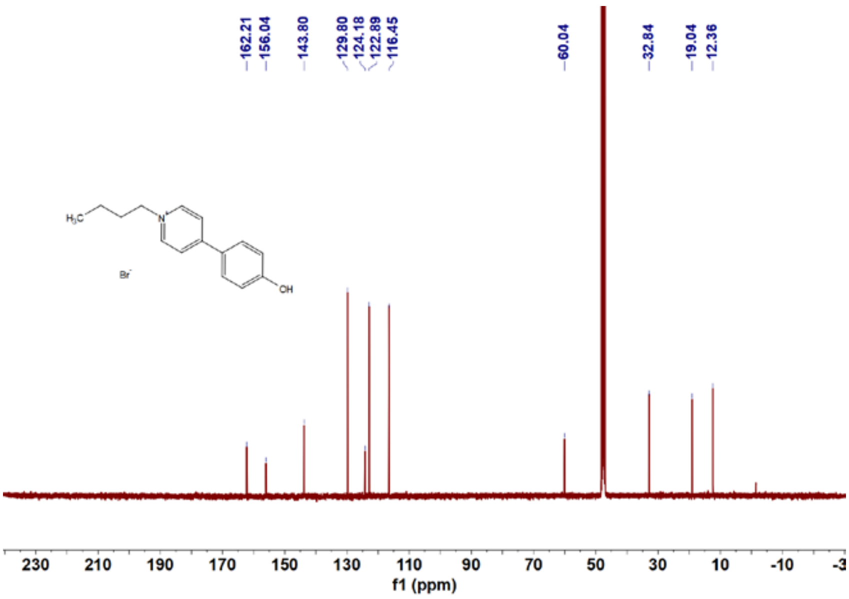

Fig. 33 ^{13}C NMR spectrum of compound R3 in methanol-d_4 (25°C).

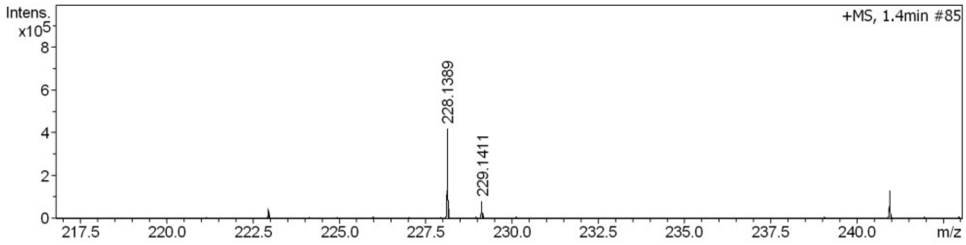

Fig. 34 ESI-HRMS spectrum of compound R3.

2. Structural characterizations, optical measurements and cell experiments.

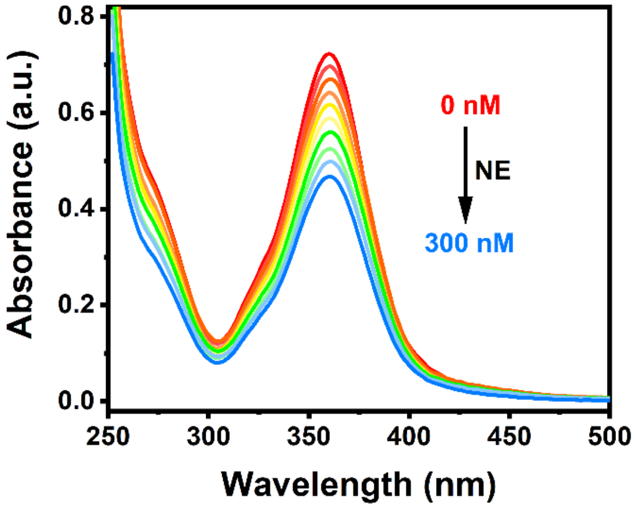

Fig. 35 UV-vis spectra of BPS3 (5 μM) upon addition of NE (0–300 nM).

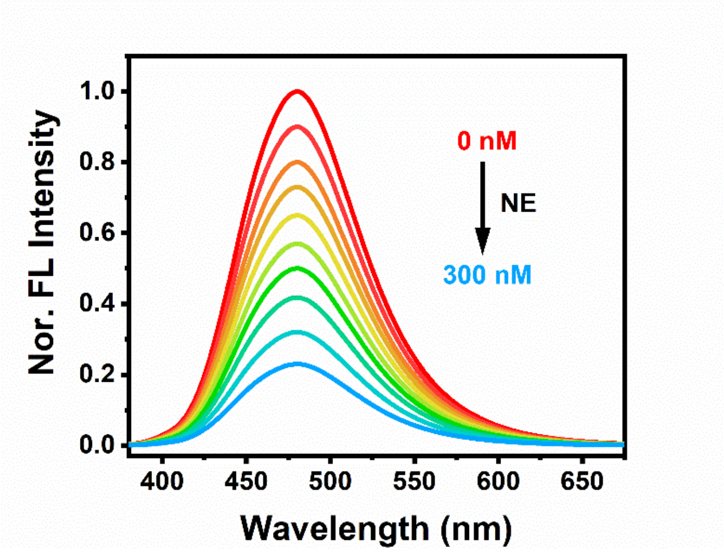

Fig. 36 Fluorescence spectra of BPS3 (5 μM) upon addition of NE (0–300 nM).

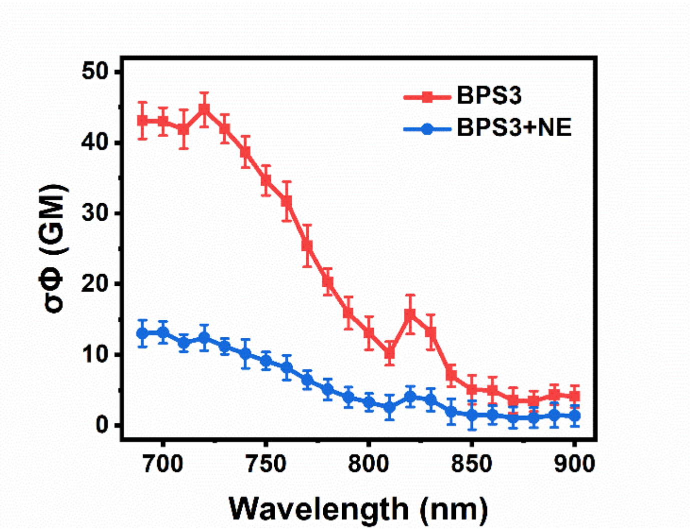

Fig. 37 Two-photon absorption (TPA) cross-section values of 5 μM BPS3 in the absence and in the presence of NE (Reference: Rhodamine B). Data are presented as mean ± S.D. Error bars: S.D., *n* = 3 independent experiments.

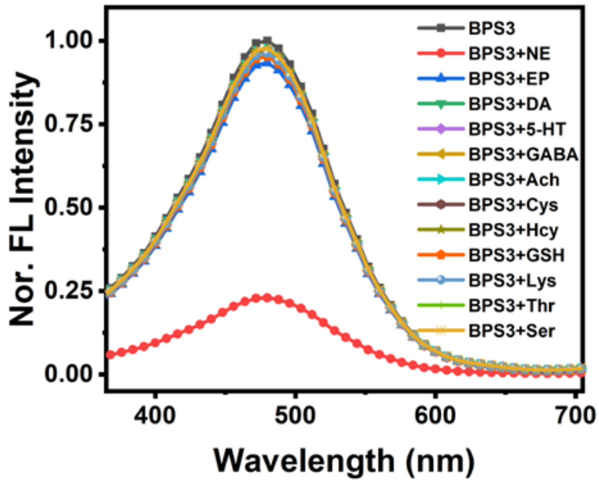

Fig. 38 Fluorescence spectra of BPS3 (5 μM) upon addition of different analytes, measured after ten minutes of mixing. The concentrations of NE, EP, and DA were 300 nM, and other species were all 1 mM, two-photo excited at 720 nm.

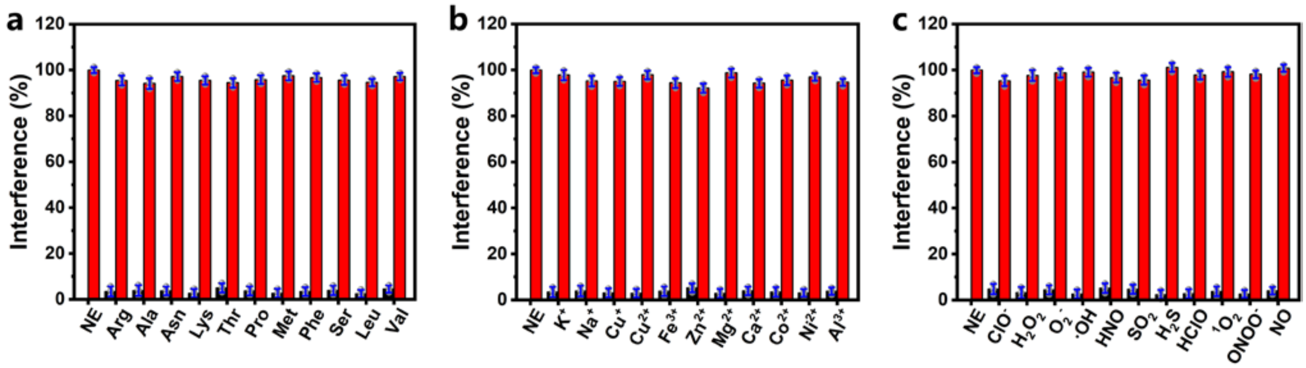

Fig. 39 The competition tests for BPS3 toward NE (300 nM) against amino acids (a), metal ions (b) and related biological species (c). The black bars represent the signal of BPS3 toward these interferents, and the red bars represent the responses of the probe toward the mixture of NE (300 nM) and potential interferents. The concentration of metal ions (except K^+, Na^+ and Cu^{2+}) and related active substances were 300 μM, while the concentrations of K^+, Na^+ and Cu^{2+} were 50 mM, 100 mM and 10 μM, respectively. Data are presented as mean ± S.D. Error bars: S.D., *n* = 3 independent experiments.

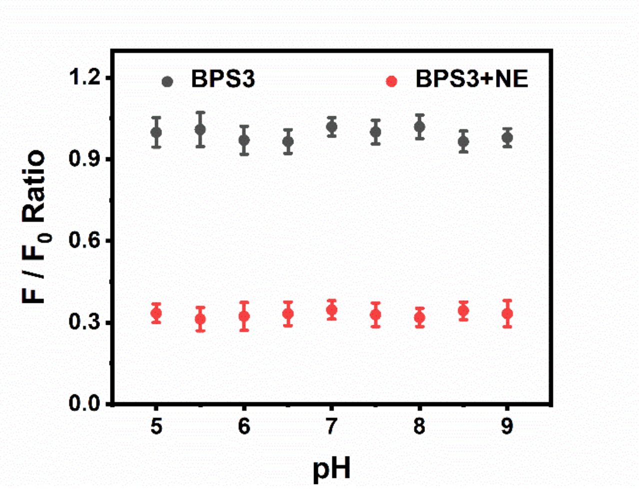

Fig. 40 The effect of pH (5.0~9.0) on the fluorescence intensity at 480 nm of BPS3 and BPS3+NE. Data are presented as mean ± S.D. Error bars: S.D., *n* = 3 independent experiments.

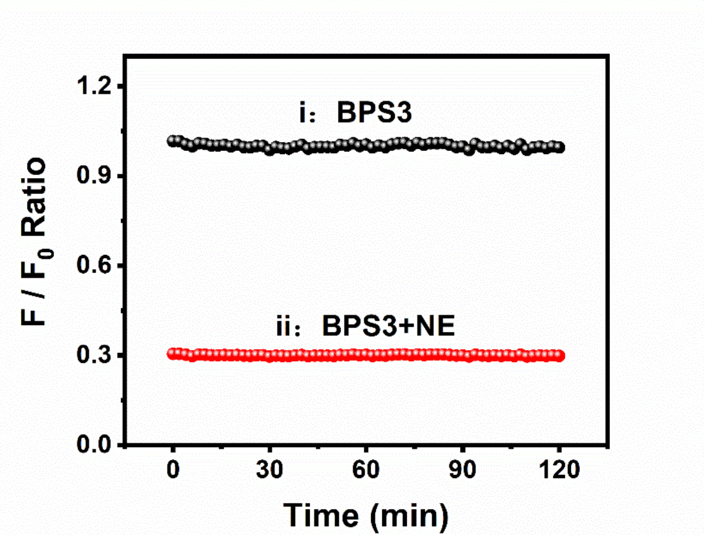

Fig. 41 Photostability test of the probe BPS3 and BPS3+NE in PBS (pH 7.4).

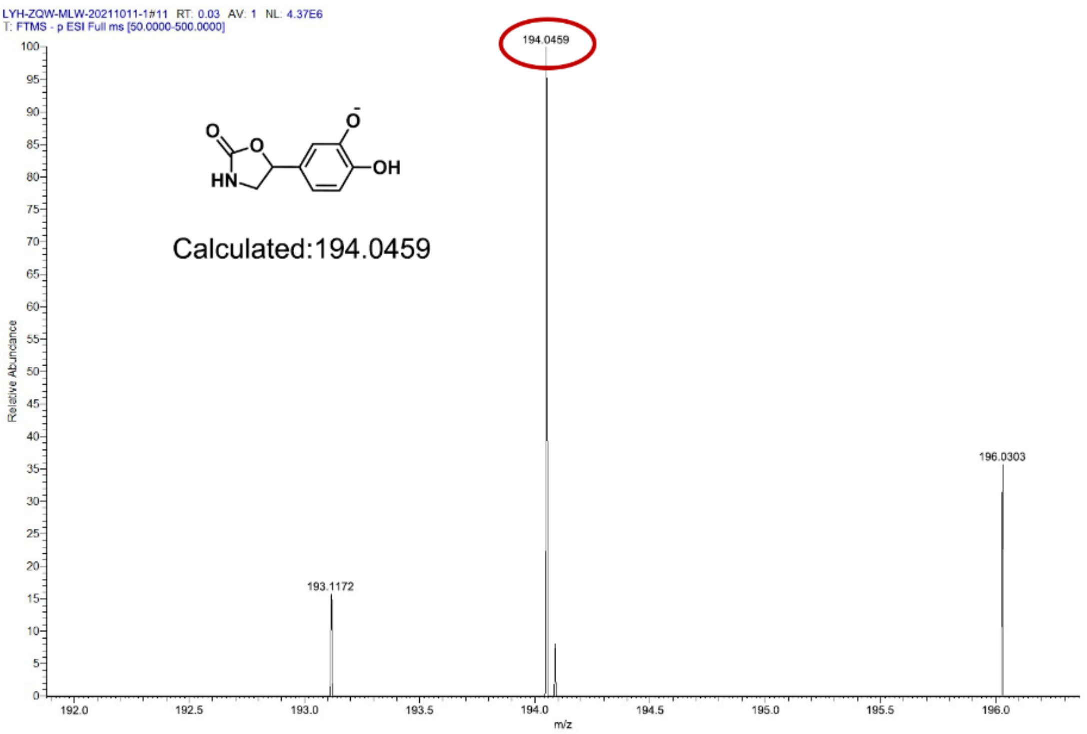

Fig. 42 ESI-HRMS spectrum of BPS3 with NE.

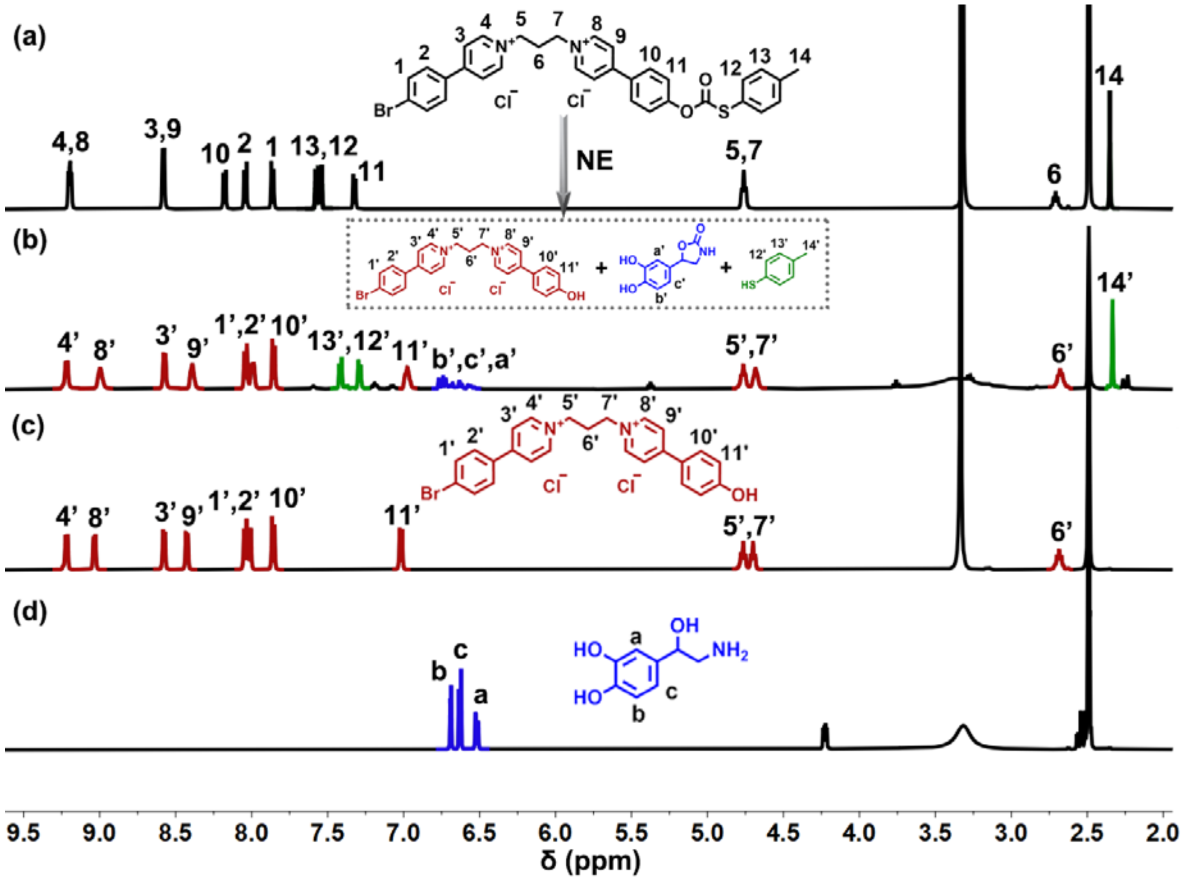

Fig. 43 ^{1}H NMR spectra of BPS3 (a), BPS3 reacted with NE for 60 mins (b), BPS3-OH (c), and NE (d), in DMSO-d_6, at 298 K.

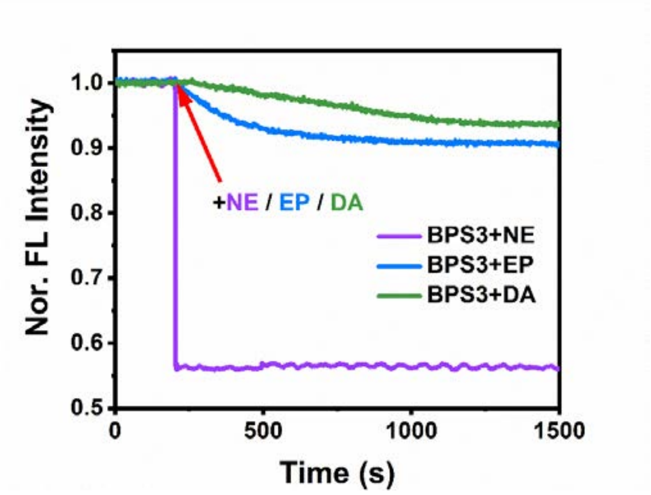

Fig. 44 Normalized fluorescence response dynamics (recorded at 480 nm) of 5 μM aqueous solution of BPS3 with addition of NE, EP, or DA (100 nM for each).

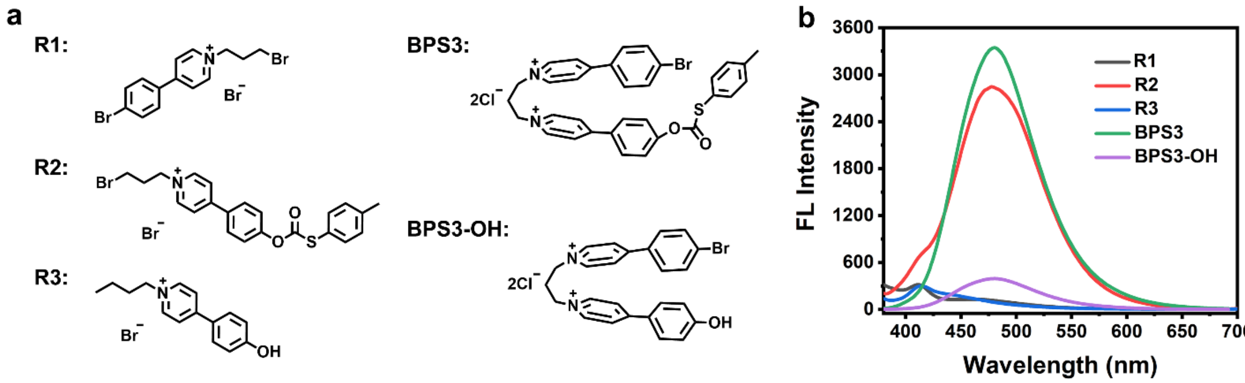

Fig. 45 (a) Chemical structures of the referred compounds. (b) Fluorescence spectra of the corresponding compounds in panel (a), 10 μM in PBS (10 mM, pH=7.4), excited at 360 nm.

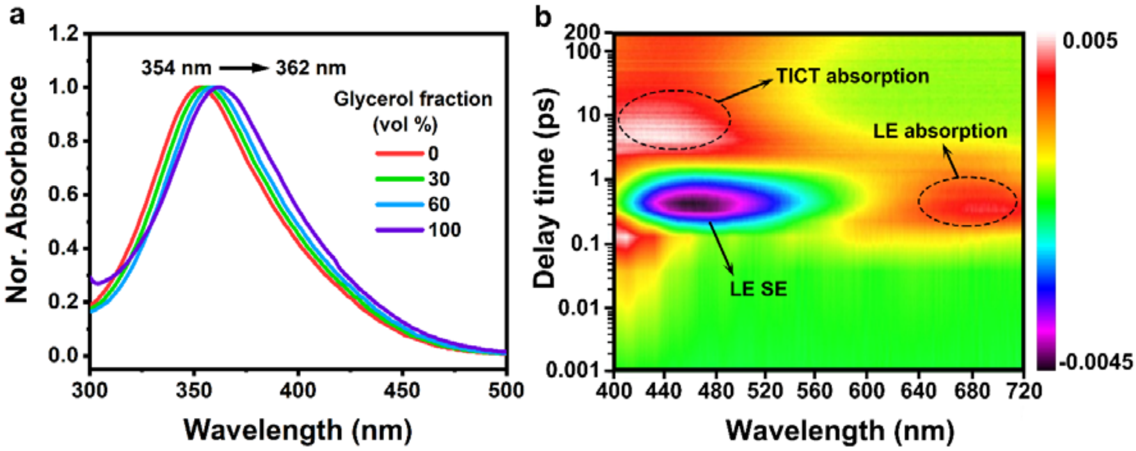

Fig. 46 (a) Viscosity-dependent normalized absorption spectra of BPS3-OH (10 μM). (b) Transient absorption spectrum of BPS3-OH, 1 mM in PBS (10 mM, pH=7.4). The color bar showed the relative optical density.

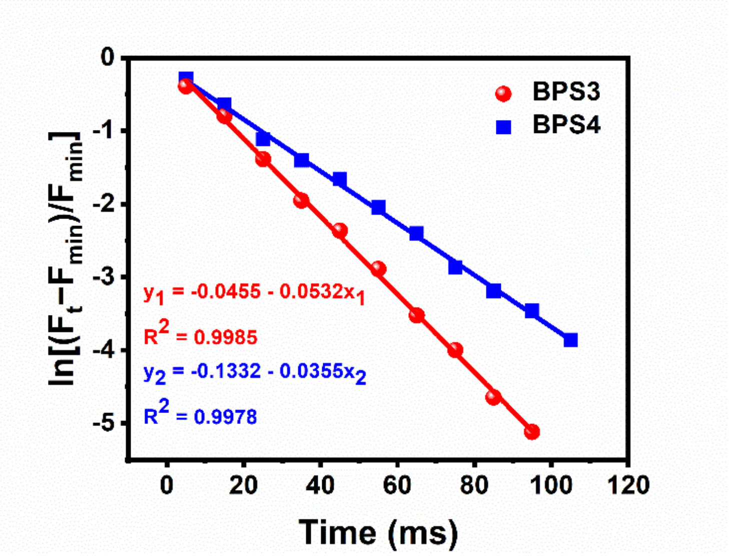

Fig. 47 Kinetic fluorescence responses of 5 μM BPS3 (red) and BPS4 (blue) to 100 nM NE in aqueous solution. The pseudo-first-order rate constants (k_{obs}) were obtained from the slope of the plot of ln [(F_t-F_{min})/F_{min}] vs time. F_{min} is the minimum fluorescence intensity during the measurement time, F_t is the fluorescence intensity of BPS3 and BPS4 at 480 nm at the corresponding time points.

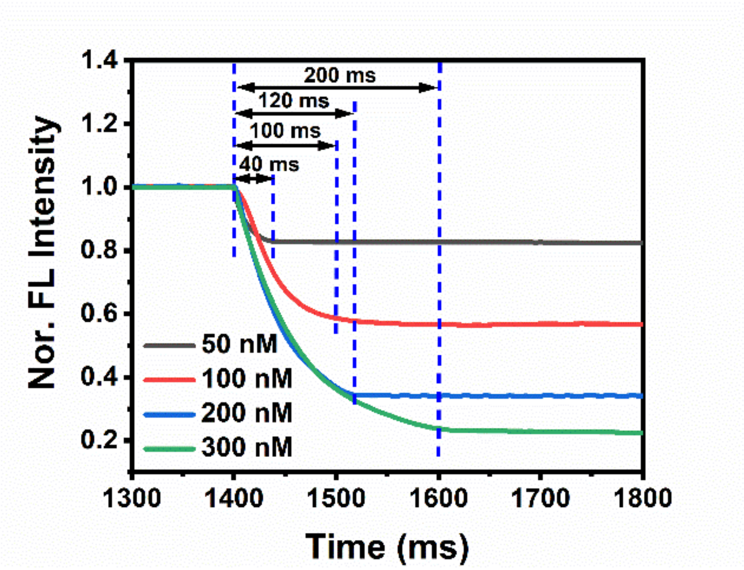

Fig. 48 Normalized fluorescence response dynamics (recorded at 480 nm) of 5 μM aqueous solution of BPS3 with addition of various concentrations of NE (50, 100, 200, and 300 nM).

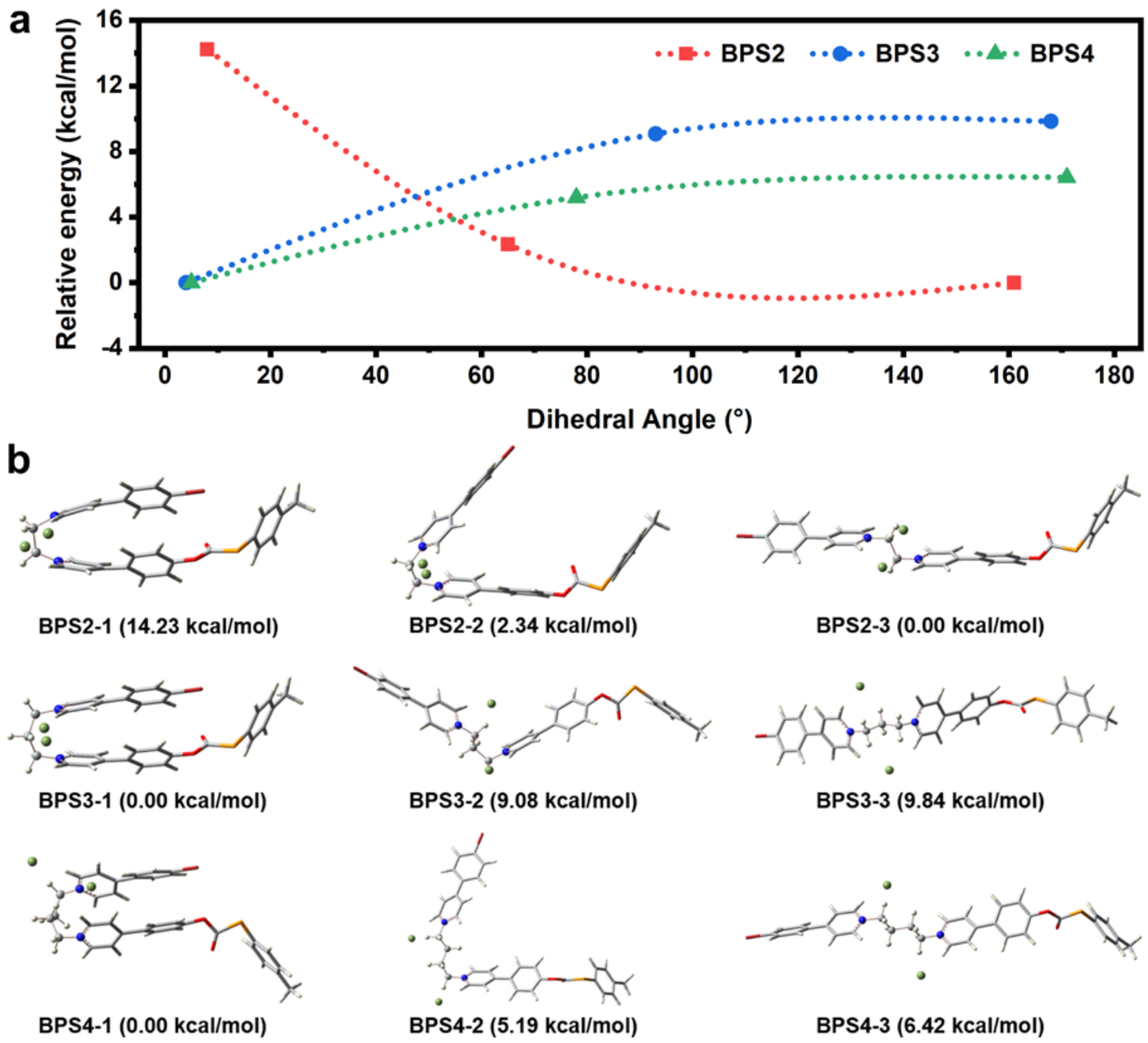

Fig. 49 (a) Relative energy in different conformation states of BPS2, BPS3 and BPS4. (b) The corresponding geometries

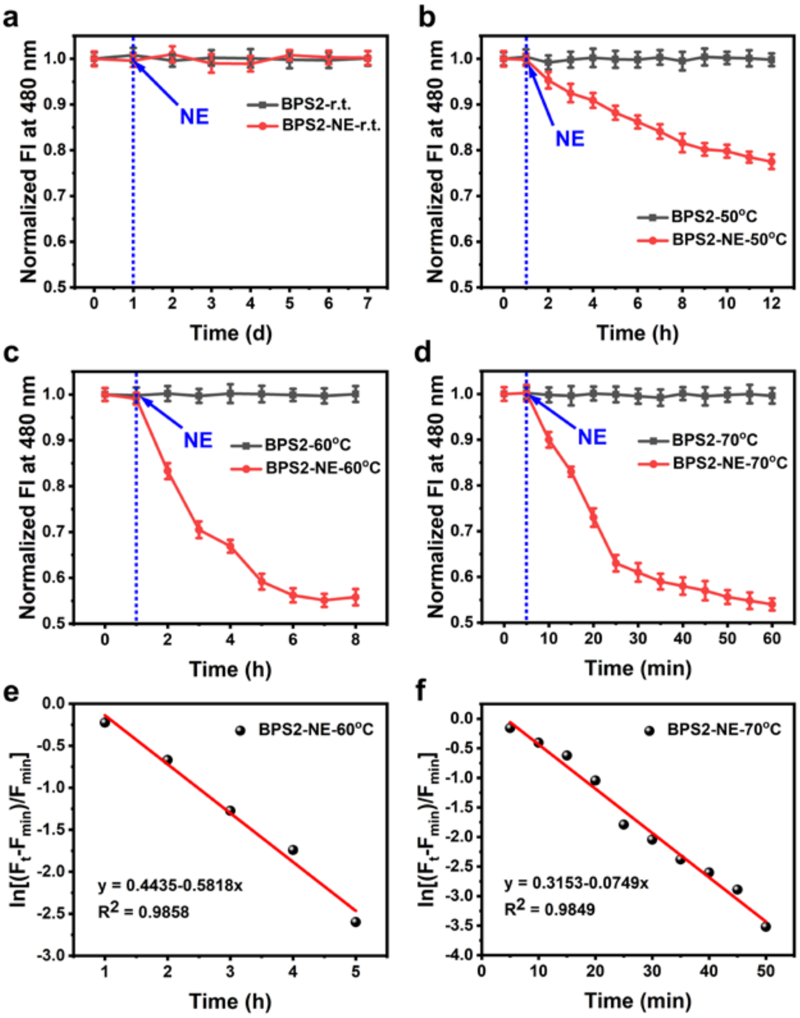

Fig. 50 Fluorescence spectra of BPS2 (5 μM) reacted with NE (100 nM) at room temperature (a), 50 °C (b), 60 °C (c) and 70 °C (d), respectively (Data are presented as mean ± S.D. Error bars: S.D., *n* = 3 independent experiments.). Kinetic fluorescence responses of 5 μM BPS2 to 100 nM NE in aqueous solution at 60 °C (e) and 70 °C (f). The pseudo-first-order rate constants (k_{obs}) were obtained from the slope of the plot of ln [(F_t-F_{min})/F_{min}] vs time. F_{min} is the minimum fluorescence intensity during the measurement time, F_t is the fluorescence intensity of BPS2 at 480 nm at the corresponding time points.

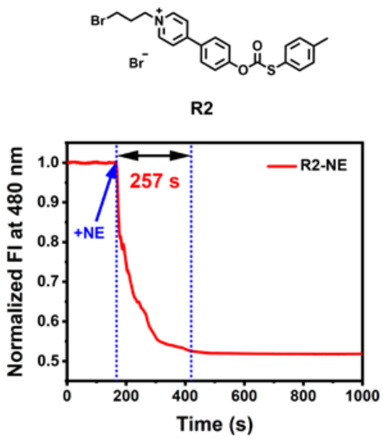

Fig. 51 Chemical structure and normalized fluorescence response dynamics of 5 μM aqueous solution of R2 toward NE.

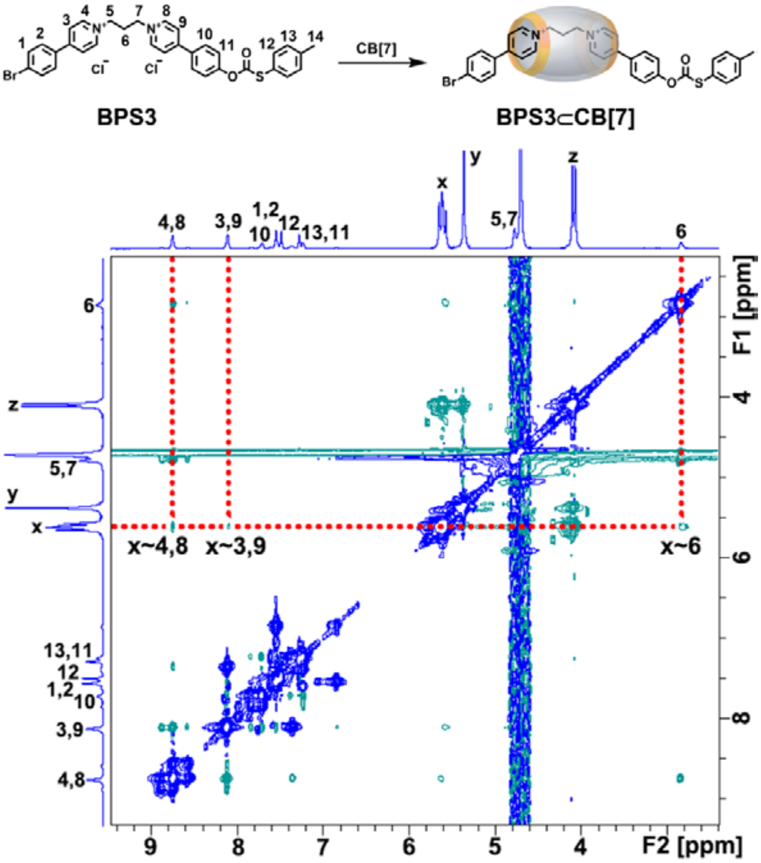

Fig. 52 Partial 2D ROESY NMR spectrum of the complex BPS3⊂CB[7], in which [BPS3] = [CB[7]] = 1 mM, in D_2O at 298 K.

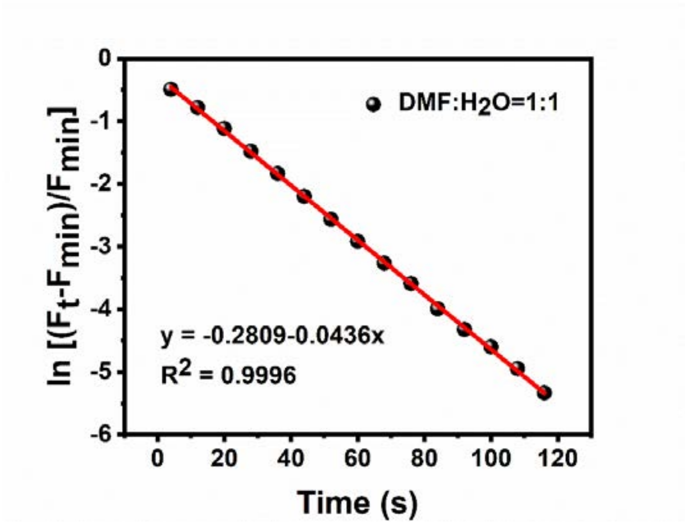

Fig. 53 Kinetic fluorescence responses of 5 μM BPS3 to 100 nM NE in DMF:H_2O (1:1). The pseudo-first-order rate constants (k_{obs}) were obtained from the slope of the plot of ln [(F_t-F_{min})/F_{min}] vs time. F_{min} is the minimum fluorescence intensity during the measurement time, F_t is the fluorescence intensity of BPS3 at 480 nm at the corresponding time points.

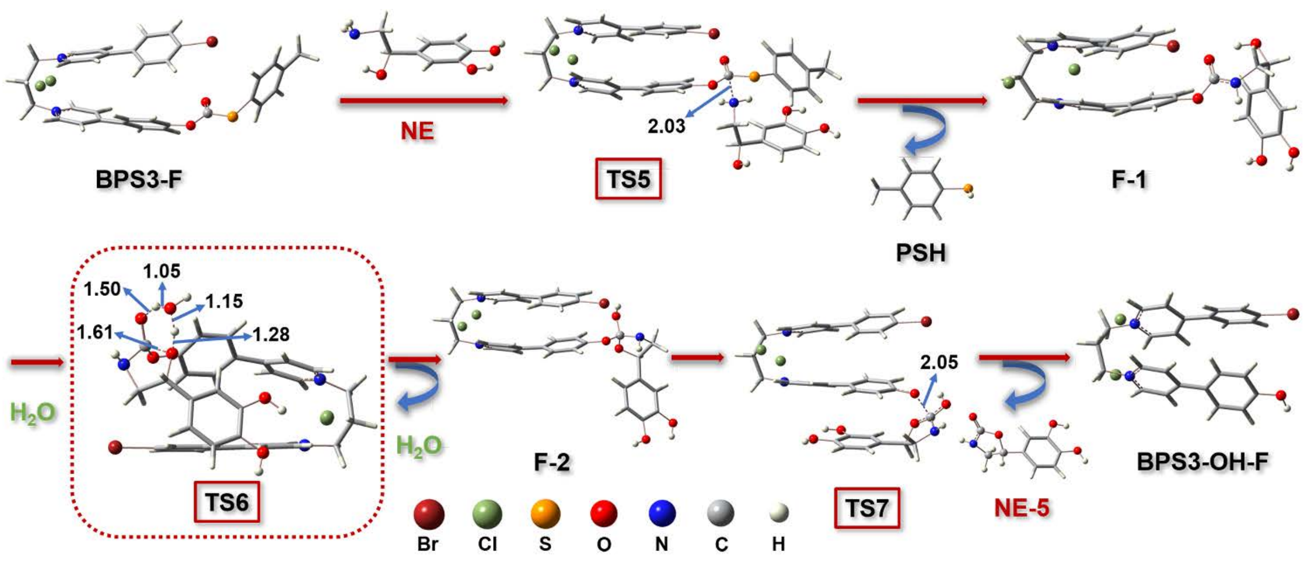

Fig. 54 The possible transition states geometries during the reactions between the folded probe BPS3 and NE with involvement of H_2O.

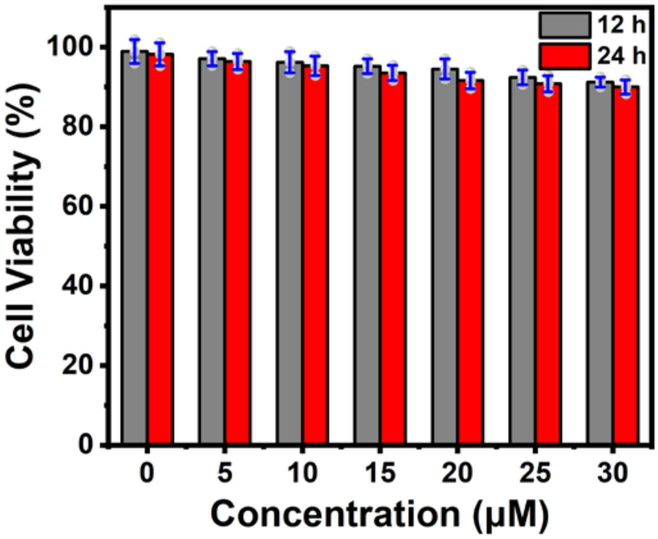

Fig. 55 MTT assay of probe BPS3 at concentrations of 0, 5, 10, 15, 20, 25 and 30 μM in neurons for 12 h (gray bars) and 24 h (red bars), respectively. Data are presented as mean ± S.D. Error bars: S.D., n = 3 independent experiments.

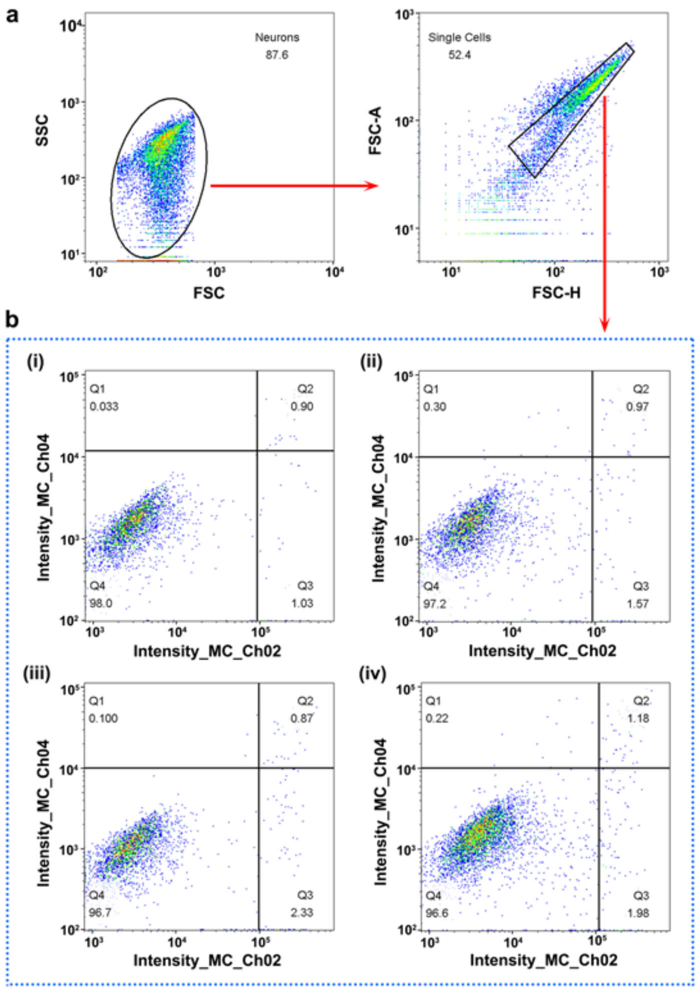

Fig. 56 (a) Representative FACS gating strategy of neurons sorting. (b) Apoptosis assay of neurons after incubated with BPS3 at concentrations of (i) 0, (ii) 10, (iii) 20, (iv) 30 μM for 24 h. Q1, Q2, Q3 and Q4 represent the regions of dead cells, late apoptotic cells, early apoptotic cells, and live cells respectively.

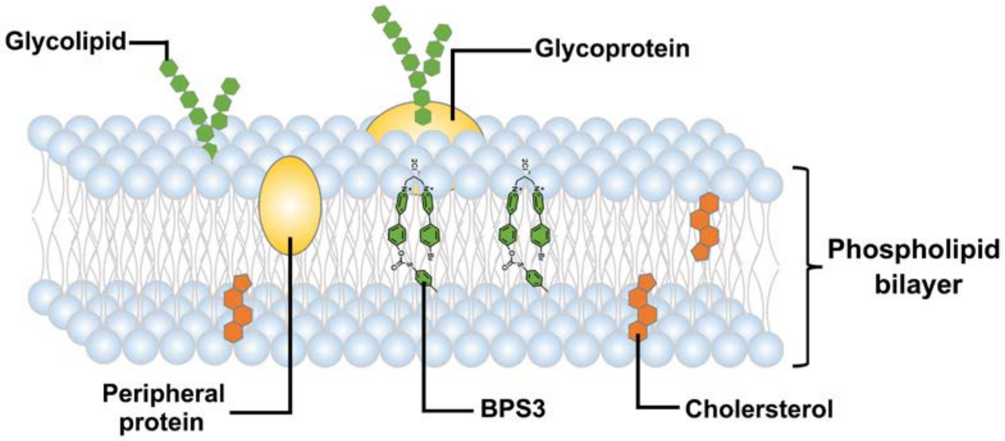

Fig. 57 Schematic representation of BPS3 targeting to cell membranes.
