# Supplementary material for: Two-photon fluorescence imaging and specifically biosensing of norepinephrine on a 100-ms timescale
Source: Nat Commun. 2023 Mar 14;14:1419. doi: 10.1038/s41467-023-36869-3 (PMC10014876; doi:10.1038/s41467-023-36869-3)
Supplement: Supplementary file 3 — Reporting Summary [file 41467_2023_36869_MOESM3_ESM.pdf]

nature portfolio

Corresponding author(s): Qi-Wei Zhang, and Yang Tian

Last updated by author(s): Feb 14, 2023

Reporting Summary

Nature Portfolio wishes to improve the reproducibility of the work that we publish. This form provides structure for consistency and transparency in reporting. For further information on Nature Portfolio policies, see our Editorial Policies and the Editorial Policy Checklist.

Statistics

For all statistical analyses, confirm that the following items are present in the figure legend, table legend, main text, or Methods section.

n/a	Confirmed	
☐	☒	The exact sample size (*n*) for each experimental group/condition, given as a discrete number and unit of measurement
☐	☒	A statement on whether measurements were taken from distinct samples or whether the same sample was measured repeatedly
☐	☒	The statistical test(s) used AND whether they are one- or two-sided<br>*Only common tests should be described solely by name; describe more complex techniques in the Methods section.*
☒	☐	A description of all covariates tested
☐	☒	A description of any assumptions or corrections, such as tests of normality and adjustment for multiple comparisons
☐	☒	A full description of the statistical parameters including central tendency (e.g. means) or other basic estimates (e.g. regression coefficient) AND variation (e.g. standard deviation) or associated estimates of uncertainty (e.g. confidence intervals)
☐	☒	For null hypothesis testing, the test statistic (e.g. *F*, *t*, *r*) with confidence intervals, effect sizes, degrees of freedom and *P* value noted<br>*Give P values as exact values whenever suitable.*
☒	☐	For Bayesian analysis, information on the choice of priors and Markov chain Monte Carlo settings
☒	☐	For hierarchical and complex designs, identification of the appropriate level for tests and full reporting of outcomes
☐	☒	Estimates of effect sizes (e.g. Cohen's *d*, Pearson's *r*), indicating how they were calculated

Our web collection on statistics for biologists contains articles on many of the points above.

Software and code

Policy information about availability of computer code

Data collection	All calculations have been performed using the DFT method implemented in the commercial Gaussian 16 program package. Molecular geomtetries of the model complexes were optimized applying the PBE0-D3BJ functional with the SMD solvation model and water as the solvent. The Stuttgart-Dresden Stuttgart-Dresden (SDD) effective core potential (ECP) was used to describe Br. For all the other atoms the 6-31G* Pople basis set was used. All of the optimized geometries mentioned were built by Gaussview 6.0. The independent gradient model based on Hirshfeld partition (IGMH) was completed by using the Multiwfn (3.8 dev) program. Flow cytometry data was analyzed by FlowJo (X 10.0.7 R2).
Data analysis	No software was used.

For manuscripts utilizing custom algorithms or software that are central to the research but not yet described in published literature, software must be made available to editors and reviewers. We strongly encourage code deposition in a community repository (e.g. GitHub). See the Nature Portfolio guidelines for submitting code & software for further information.

Data

Policy information about availability of data

All manuscripts must include a data availability statement. This statement should provide the following information, where applicable:

- Accession codes, unique identifiers, or web links for publicly available datasets
- A description of any restrictions on data availability
- For clinical datasets or third party data, please ensure that the statement adheres to our policy

All data supporting the findings of this study are available in the main text and the Supplementary Information.

Human research participants

Policy information about studies involving human research participants and Sex and Gender in Research.

Reporting on sex and gender	Not relevant to this study.
Population characteristics	Not relevant to this study.
Recruitment	Not relevant to this study.
Ethics oversight	Not applicable.

Note that full information on the approval of the study protocol must also be provided in the manuscript.

Field-specific reporting

Please select the one below that is the best fit for your research. If you are not sure, read the appropriate sections before making your selection.

☒ Life sciences ☐ Behavioural & social sciences ☐ Ecological, evolutionary & environmental sciences

For a reference copy of the document with all sections, see nature.com/documents/nr-reporting-summary-flat.pdf

Life sciences study design

All studies must disclose on these points even when the disclosure is negative.

Sample size	Sample size was not applied in the study, which did not involve statistical analysis. We used similar sample sizes to the literatures in the field. "A genetically encoded fluorescent sensor enables rapid and specific detection of dopamine in flies, fish, and mice." Cell, 174(2), 481-496. "A genetically encoded fluorescent sensor for rapid and specific in vivo detection of norepinephrine." Neuron 102.4 (2019): 745-761. "Spatiotemporal dynamics of noradrenaline during learned behaviour." Nature 606, (2022): 732-738.
Data exclusions	No data were excluded from the analyses
Replication	All the experiments were repeated at least three times to make sure the results are repeatable.
Randomization	Cells in mouse brain tissue slices were randomly assigned into control or experimental groups.
Blinding	The investigators were not blind to group allocation during data collection and analysis. The experimental conditions were obvious to the researchers and the analysis were performed objectively and not subjective to human bias.

Reporting for specific materials, systems and methods

We require information from authors about some types of materials, experimental systems and methods used in many studies. Here, indicate whether each material, system or method listed is relevant to your study. If you are not sure if a list item applies to your research, read the appropriate section before selecting a response.

Materials & experimental systems

n/a	Involved in the study
☒	☐ Antibodies
☒	☐ Eukaryotic cell lines
☒	☐ Palaeontology and archaeology
☐	☒ Animals and other organisms
☒	☐ Clinical data
☒	☐ Dual use research of concern

Methods

n/a	Involved in the study
☒	☐ ChIP-seq
☐	☒ Flow cytometry
☒	☐ MRI-based neuroimaging

Animals and other research organisms

Policy information about studies involving animals; ARRIVE guidelines recommended for reporting animal research, and Sex and Gender in Research

Laboratory animals	5-month-old mice (C57BL/6 for normal mice, and APP/PS1 for AD mice). The housing facility is maintained at 22 °C and 35%-55% humidity on a 12-h light/dark cycle (lights on at 8:00 am).
Wild animals	The study did not involve wild animals.
Reporting on sex	The present research did not involve sex-differentiated experiments, and were not applied to only one sex. Due to the small sample size, sex of the mouse was not considered in the study design, and information on sex of mouse was not collected in the study.
Field-collected samples	The study did not involve samples collected from the field.
Ethics oversight	Animal Care and Use Committee of East China Normal University (Shanghai, China)

Note that full information on the approval of the study protocol must also be provided in the manuscript.

Flow Cytometry

Plots

Confirm that:

☒ The axis labels state the marker and fluorochrome used (e.g. CD4-FITC).

☒ The axis scales are clearly visible. Include numbers along axes only for bottom left plot of group (a 'group' is an analysis of identical markers).

☒ All plots are contour plots with outliers or pseudocolor plots.

☒ A numerical value for number of cells or percentage (with statistics) is provided.

Methodology

Sample preparation	Newborn within 24 hours C57BL/6 wild-type mice were anesthetized with halothane, and then the whole brain tissues were removed quickly and put in Hanks' balanced salt solution (HBSS, free of Mg2+ and Ca2+) in an ice bath. Mouse cortical tissues were quickly striped and cultured in papain for 15 min at 37 °C, after that they were dispersed into poly-d-lysine-coated 35 mm Petri dishes at a density of 1 × 106 cells/dish. Neurons were cultured with neurobasal medium containing L-Glutamine and B27 (37 °C, 5% CO2, 95% O2) and the medium was changed three times a week.
Instrument	FACSCalibur flow cytometry (Becton, Dickinson and Company, USA)
Software	FlowJo (X 10.0.7 R2)
Cell population abundance	Cell population abundance was determined by comparing the stained cells with negative controls.
Gating strategy	Unstained cells were used as negative controls, while cells treated with probe at concentrations of 0, 10, 20, and 30 μM for 24 hours were used as positive controls for FITC/PI staining. The horizontal axis and the vertical axis are respectively set as FITC channel and PI channel. With reference to the control group without probe treatment, a cross gate is drawn to determine the proportion of cells in different groups. An exemplified gating strategy could be found in Supplementary Fig. 56.

☒ Tick this box to confirm that a figure exemplifying the gating strategy is provided in the Supplementary Information.
